# Fungal appressoria as mechanochemical organelles for polyurethane degradation

**DOI:** 10.1371/journal.pgen.1012292

**Published:** 2026-08-31

**Authors:** Fan Fei, Zhenjie Su, Rui Liu, Rongrong Gao, Chaomin Sun

**Affiliations:** 1 Laboratory of Experimental Marine Biology & Center of Deep Sea Research, Shandong Province Key Laboratory of Marine Biodiversity and Bio-resource Sustainable Utilization, Institute of Oceanology, Chinese Academy of Sciences, Qingdao, China; 2 Marine Academy of Zhejiang Province, Hangzhou, China; 3 Laboratory for Marine Biology and Biotechnology, Qingdao Marine Science and Technology Center, Qingdao, China; 4 College of Earth Science, University of Chinese Academy of Sciences, Beijing, China; 5 Key Laboratory of Ocean Space Resource Management Technology, Ministry of Natural Resources, Hangzhou, China; 6 Yantai Vocational College, Yantai, China; Australian National University Research School of Biology, AUSTRALIA

## Abstract

Polyurethane (PU) is a synthetic polymer characterized by highly stable urethane linkages that hinder biological turnover. Although fungi have been implicated in PU degradation, the molecular mechanisms that couple polymer surface sensing to enzymatic depolymerization remain largely undefined. Here we show that the marine-derived fungus *Alternaria alternata* FB1 employs a surface-sensing signaling pathway that drives appressorium-mediated degradation of both polyester and polyether PUs. Contact with hydrophobic polymer surfaces rapidly induces melanized appressoria that mechanically penetrate the polymer matrix and promote its oxidative and hydrolytic depolymerization. Integrative transcriptomic analysis and targeted gene disruption identify the mucin-like surface sensor Msb2 as an upstream component of the polyurethane surface-sensing machinery. Loss of Msb2 disrupts MAPK and Ca^2+^ signaling, impairs appressorium differentiation, and reduces expression of degradative enzymes. Biochemical profiling further reveals multiple urethane-hydrolyzing enzymes that expand the known catalytic repertoire for PU bond cleavage. Together, these findings establish a mechanistic framework linking surface recognition, appressorium development, and polymer degradation, providing insight into how fungi transform recalcitrant polyurethane materials.

## Author summary

Plastics are now found in nearly every environment on Earth, yet most are extremely difficult for living organisms to break down. Polyurethane is one such plastic and is widely used in foams, coatings, and adhesives. While some fungi have been reported to degrade plastics, we still know little about how these organisms recognize synthetic materials and activate the cellular processes needed to transform them. In this study, we investigated how a marine fungus responds when it encounters polyurethane surfaces. We found that the fungus forms specialized structures normally used to penetrate plant tissues. These structures allow the organism to tightly attach to the plastic surface and concentrate enzymes that break chemical bonds within the material. Using genetic and transcriptomic approaches, we identified a membrane protein that acts as a sensor for hydrophobic surfaces and triggers a signaling pathway controlling this response. This pathway coordinates cellular differentiation and enzyme production, enabling the fungus to interact with and degrade polyurethane. Our findings reveal how existing fungal genetic programs can be redirected to interact with synthetic materials, providing new insight into the biological mechanisms that may influence the persistence of plastics in the environment.

## Introduction

Polyurethane (PU), the sixth most produced synthetic polymer worldwide, is extensively employed across sectors such as construction, healthcare, automotive, and textiles, and its global market continues to expand [1–3]. The fundamental PU backbone comprises urethane (carbamate) linkages formed between isocyanate and polyol monomers. Depending on the polyol type, PU is classified into polyester polyurethane (PAUR) and polyether polyurethane (PEUR). PAUR, characterized by its high chemical resistance and flexibility, is commonly used in coatings and synthetic fibers, whereas PEUR, owing to its superior hydrolytic stability and resistance to aging, is widely applied in building insulation and electronic consumer goods [4]. However, recycling and disposal of PU waste remain a formidable challenge. Conventional routes such as landfilling, incineration, mechanical, and chemical recycling are energy-intensive and prone to secondary pollution [5]. In contrast, biodegradation offers an environmentally benign alternative under mild conditions, preserving much of the polymer’s chemical complexity and enabling recovery of monomeric units for value-added reuse [6]. A detailed understanding of the key biological processes underlying PU degradation is therefore critical for the development of sustainable waste-management strategies.

Filamentous fungi are increasingly recognized as important agents of PU degradation owing to their diverse repertoire of extracellular enzymes and their capacity for mechanical penetration [7]. Several fungal genera, including *Aspergillus*, *Trichoderma*, *Penicillium*, and *Fusarium*, have been documented to degrade PU [8–10]. It is generally held that hyphal tips physically breach PU surfaces, thereby increasing substrate accessibility for enzymatic hydrolysis, and that secreted enzymes act synergistically to accelerate depolymerization. Nevertheless, mechanisms by which fungi recognize polyurethane surfaces, initiate substrate colonization, and coordinate degradative responses remain poorly understood. Of particular interest is the appressorium, a morphogenetic structure extensively characterized by its roles in surface recognition, adhesion, and penetration in phytopathogenic fungi [11–13].

The appressorium is a specialized infection structure that generates extraordinary turgor pressure (up to 8.0 MPa) to mechanically breach host surfaces [12]. Studies in phytopathogenic fungi *Magnaporthe oryzae* have shown that appressorium development is controlled by conserved signaling pathways, particularly the mitogen-activated protein kinase (MAPK), cyclic AMP-protein kinase A (cAMP-PKA), and oxidative-stress response pathways. Members of the genus *Alternaria* likewise possess strong surface-colonization capabilities and readily differentiate appressoria in response to environmental cues [11,14]. In *A. alternata*, appressorium formation is regulated by phospholipase C-mediated Ca^2+^ signaling and reactive oxygen species (ROS) dynamics [15–17]. This morphogenetic transition is frequently coupled with secretion of cell-wall-degrading enzymes [18], raising the possibility that pathways governing fungal invasion of biological substrates may also facilitate degradation of synthetic polymers.

In this study, we investigated the mechanisms underlying polyurethane degradation by the marine fungus *A. alternata* strain FB1 and examined how polyurethane surfaces are recognized and how surface sensing is coupled to appressorium differentiation and polymer degradation. Our findings reveal a regulatory framework linking hydrophobic surface perception, appressorium development, and enzymatic depolymerization, providing new insights into fungal plastic biodegradation.

## Results

### Biodegradation of PU by the marine fungus *A. alternata* FB1

**Fig 1:**
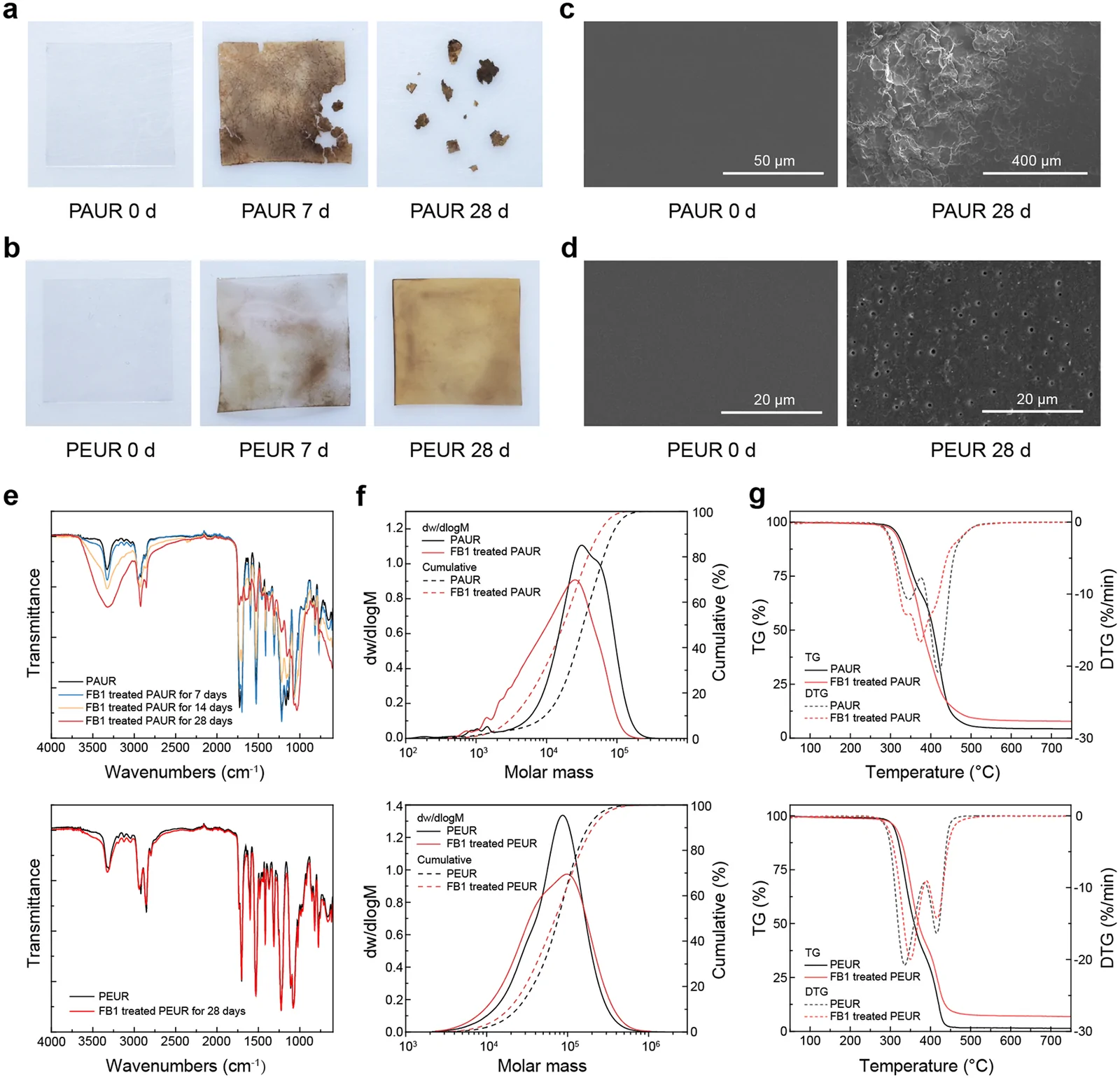
Degradation of polyurethane by *A. alternata* FB1. a, b) Degradation of polyester polyurethane (PAUR) and polyether polyurethane (PEUR) films by the marine fungus *A. alternata* FB1 over different incubation periods. c, d) SEM images of PAUR and PEUR films before and after colonization by strain FB1. e) FTIR spectra of PAUR and PEUR films following strain FB1 treatment. f) Molecular weight distribution of PAUR and PEUR films after 28 days of treatment by strain FB1. g) Thermogravimetric (TG) and derivative thermogravimetric (DTG) curves of PAUR and PEUR films after 28 days of treatment by strain FB1.

To elucidate the degradative action of *A. alternata* FB1, polyester polyurethane (PAUR) and polyether polyurethane (PEUR) films were overlaid onto the surface of strain FB1 cultured on rice-based solid medium. Following co-incubation for 7–28 days, the films were retrieved for morphological examination and physicochemical parameter analysis. After 28 days of incubation, PAUR films were largely degraded, leaving only minor fragments. In contrast, PEUR exhibited a lower degree of degradation, with the films turning brownish post-incubation and showing no significant change in extensibility (Fig 1a, 1b). Scanning electron microscopy (SEM) revealed a flaked, layered debris pattern on PAUR film surfaces, whereas colonized PEUR films displayed dense pore formation with pore diameters of approximately 1 ~ 3 µm (Fig 1c, 1d).

After fungal treatment, both PAUR and PEUR films showed the emergence of new hydroxyl characteristic absorption peaks on their surfaces, with PAUR films exhibiting more pronounced alterations in surface functional groups (Fig 1e). These changes are consistent with oxidative and/or hydrolytic modification of the polymer surface, resulting in increased abundance of hydrophilic functional groups. Gel permeation chromatography (GPC) analysis demonstrated that after 28 days of fungal exposure, the number-average molecular weight (Mn) of PAUR films decreased from 11,548–7,170, while the weight-average molecular weight (Mw) declined from 42,309–20,865, corresponding to reductions of 37.91% and 50.68%, respectively (Fig 1f). These molecular-weight reductions are consistent with extensive polymer-chain cleavage and the accumulation of lower-molecular-weight products. By comparison, PEUR films exhibited less pronounced molecular weight changes: the proportion of fractions below 40,000 increased from 22.45% to 29.29%, and the fraction above 200,000 increased by 3.96%, suggesting a preferential degradation of mid- to short-chain segments in PEUR.

In terms of thermal performance, thermogravimetric analysis (TGA) revealed that the main degradation peak of PAUR shifted from 419 °C to 361 °C, accompanied by reduced mass loss in 400 ~ 500 °C (Fig 1g), indicating a decrease in heat-resistant components following ester bond hydrolysis and an overall reduction in thermal stability [19]. In contrast, PEUR films exhibited an initial degradation peak shift from 332 °C to 351 °C, along with a slower degradation rate and greater residual mass, suggesting that fungal treatment altered the relative abundance of thermally distinct polymer fractions. Differential scanning calorimetry (DSC) further demonstrated that the endothermic peak corresponding to the glass transition temperature of the PEUR soft segments (approximately 89 °C) was significantly attenuated (S1 Fig), consistent with reduced cross-link density and depolymerization of polymer chains. Collectively, PAUR and PEUR exhibited distinct physicochemical responses following fungal treatment, indicating that the two polyurethane types undergo different degradation trajectories.

Analysis of the degradation products from fungal-treated polyurethane subsequently revealed monomeric compounds such as butanediol, 4,4’-methylenedianiline (MDA), and the antioxidant 2246 in both PAUR and PEUR samples (S2 Fig). These findings support the conclusion that *A. alternata* FB1 cleaves the principal chemical bonds within the polymer matrix, releasing synthesis monomers and associated additives. Additionally, the detection of hexyl cyclohexanecarboxylate, which has also been reported in polyurethane degradation by other microorganisms such as *Alicycliphilus denitrificans* [20], suggests that different microbial taxa may share analogous degradation pathways or enzymatic mechanisms, thereby providing critical insights into the microbial degradation mechanisms of polyurethane.

To identify genes involved in polyurethane degradation, RNA-seq was performed on mycelia directly contacting PU films for 24 h, using adjacent surface mycelia not covered by the films as controls (three biological replicates per group). Comparative transcriptomic analysis revealed a coordinated enzymatic response underpinning polyurethane degradation (S3 Fig). We observed significant upregulation of 13 ester hydrolases (predominantly cutinases and esterases) implicating them as candidate catalysts of ester bond cleavage. Simultaneously, 20 amide-bond-cleaving enzymes, including amidases, proteases, and peptidases, were broadly induced, suggesting a potential role in urethane bond hydrolysis. In parallel, 13 extracellular oxidases exhibited marked expression changes, suggesting that oxidative modification of the polymer surface introduces polar groups that enhance hydrolase accessibility and activity.

### PU contact triggers appressoria-mediated perforation

**Fig 2:**
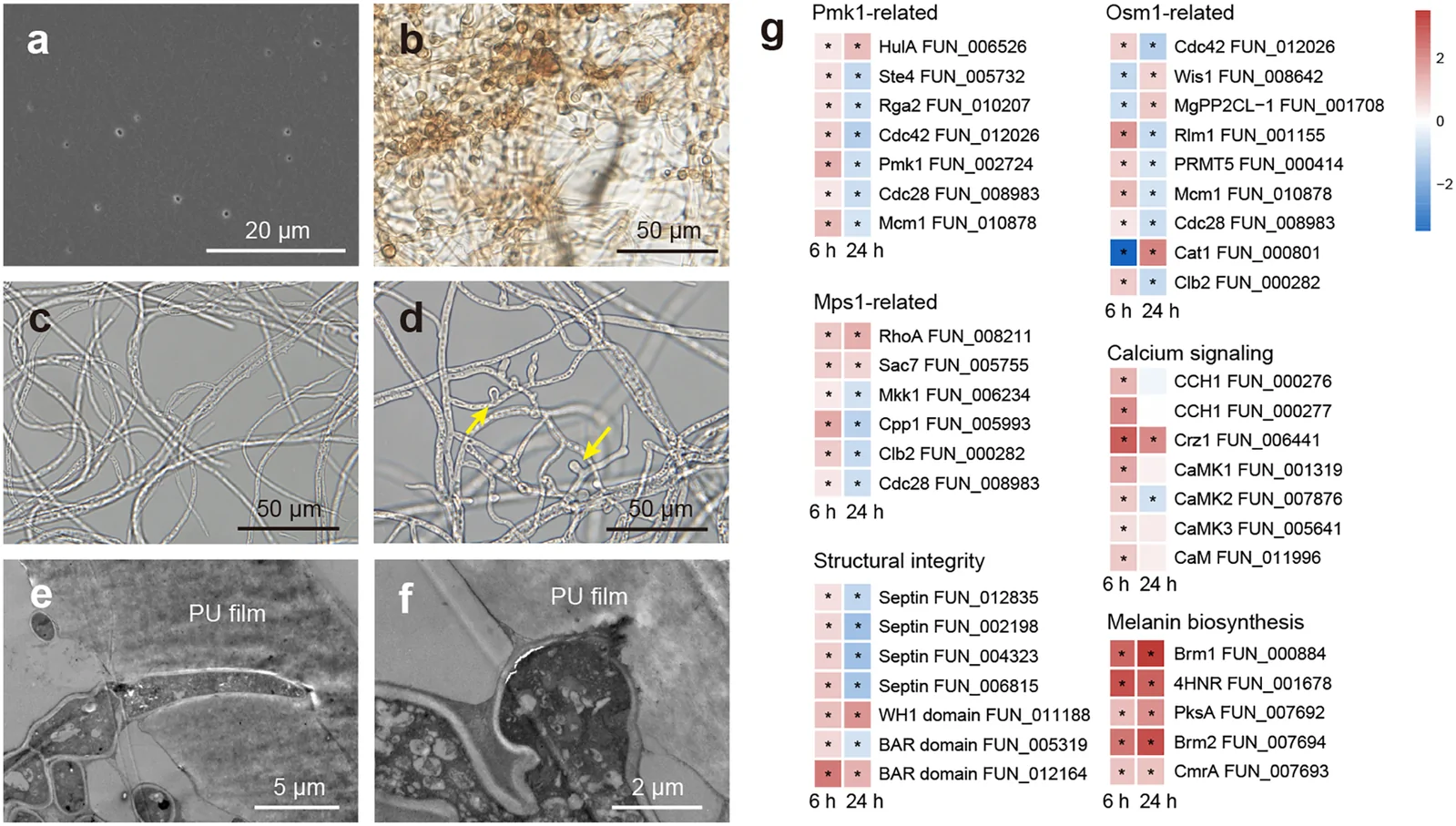
Appressoria may be involved in the degradation of polyurethane by *A. alternata* FB1. a) SEM image of PEUR film after 24 h of treatment by *A. alternata* FB1. b) DAB-stained hyphae in contact with the PEUR surface. c) Hyphal morphology of *A. alternata* FB1 under normal growth conditions. d) Morphological alterations in hyphae of *A. alternata* FB1 following 24 h of film contact. The yellow arrows indicate the formation of appressoria structures. e, f) TEM images of hyphal ultrastructure at the interface with the polyurethane film. g) Differentially expressed genes were identified following 6 h and 24 h of co-incubation with the film. Genes exhibiting statistically significant differential expressions (*P* < 0.05) were denoted by an asterisk (*). Each experimental group included three independent biological replicates.

Surprisingly, PEUR films co-incubated with *A. alternata* FB1 for only 24 h exhibited the appearance of sparse surface pores (Fig 2a). Such pore formation has typically been attributed to localized enzymatic hydrolysis of the polymer. However, the rapid appearance of these pores suggested that enzymatic activity alone may not fully explain their formation, raising the possibility that mechanical penetration also contributes to surface disruption. Notably, hyphae in contact with PEUR films differentiated into specialized, swollen structures that stained brownish upon 3,3’-diaminobenzidine (DAB) treatment, contrasting with the morphology of hyphae under normal culture conditions (Fig 2b-2d). These structures resemble the appressoria produced by *A. alternata* during plant infection, in which ellipsoidal appressoria facilitate host penetration (e.g., on Japanese pear) [21], suggesting that contact with the PEUR surface induces appressorium formation in strain FB1.

Transmission electron microscopy (TEM) revealed that these infection structures produced peg-like hyphae capable of directly penetrating the PEUR film, forming penetration pores of approximately 1 ~ 2 µm in diameter (Fig 2e, 2f). Dark, irregular halos observed at hyphal tips in direct contact with the film may indicate localized enzymatic degradation. These observations suggest that appressoria-like structures contribute to pore formation through a combination of mechanical penetration and localized enzymatic activity. The resulting micropores may serve as localized entry points for extracellular enzymes and oxidative reactions, increasing access to internal polymer chains and thereby facilitating the progressive degradation during prolonged incubation.

Because appressoria form rapidly upon contact with solid surfaces [22], RNA-seq analysis was performed after 6 h of PEUR exposure, using adjacent non-contact mycelia as controls, to distinguish early PU recognition from later adaptive responses. At this stage, key genes in the mitogen-activated protein kinase (MAPK) signaling pathway, particularly those associated with the core kinases Pmk1, Osm1, and Mps1, were significantly upregulated (Fig 2g), indicating that MAPK activation represents an early response to PEUR surface perception [15]. In parallel, genes related to ion transport and calcium signaling, including calmodulin (CaM), the zinc-finger transcription factor Crz1, and downstream Ca^2+^/calmodulin-dependent protein kinases (CaMKs), were markedly induced, suggesting that Ca^2+^ influx participates in the initial conversion of surface cues into appressorium differentiation signals [23–25]. Concurrent upregulation of cytoskeletal genes, especially septin family members and proteins containing Bin-Amphiphysin-Rvs (BAR) or WH1 domains, further indicates membrane and cytoskeletal remodeling during the early establishment of penetration structures [26]. Melanin biosynthesis genes were also elevated at this stage and remained induced at 24 h, consistent with their role in reinforcing appressorium maturation and mechanical competence.

In contrast, genes involved in cell-cycle regulation and DNA replication displayed divergent expression patterns between 6 h and 24 h (S4 Fig), implying an early proliferative response to surface contact followed by a shift toward invasive and degradative functions. The early transcriptional response was therefore characterized mainly by signaling, Ca^2+^ mobilization, membrane remodeling, and appressorium-associated morphogenesis, whereas the later response involved broader metabolic reprogramming, stress adjustment, and preparation for degradative activity. This temporal distinction indicates that *A. alternata* FB1 first interprets the PEUR surface as a developmental cue and subsequently reallocates cellular resources toward invasion, degradation, and tolerance of polymer-associated stresses.

### Synergistic regulation of appressorium morphogenesis and PU degradation

**Fig 3:**
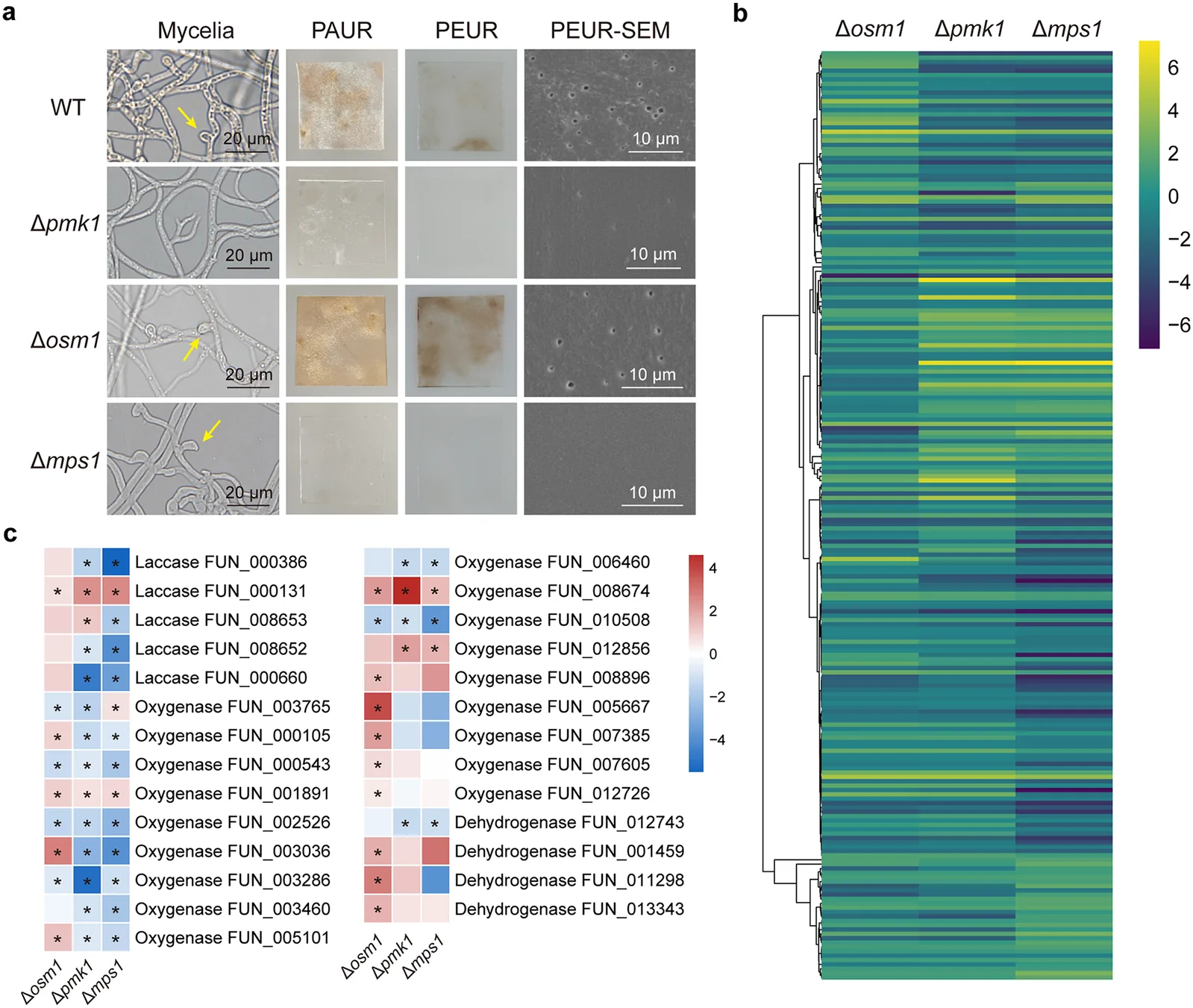
The formation of appressoria is closely related to the degradation of polyurethane by *A. alternata* FB1. a) Impact of deletion of core kinases in MAPK pathway on appressorium formation and the polyurethane degradation ability of *A. alternata* FB1. “WT” represents the wild-type *A. alternata* FB1, serving as the control. “Mycelia” depicts the hyphal morphology of different FB1 strains after 24 h of contact with PU films, with yellow arrows indicating the formation of appressoria structures. “PAUR” and “PEUR” show the macroscopic changes in PAUR or PEUR films, respectively, after three days of co-incubation with each strain. “PEUR-SEM” presents the microstructural morphology of PEUR films observed via SEM after three days of co-incubation with each strain. b) Heatmap illustrating differentially expressed genes in the Δ*pmk1*, Δ*mps1*, and Δ*osm1* mutants, primarily encompassing categories related to signal transduction, cellular architecture, cell cycle regulation, transporters, and extracellular oxidoreductases/hydrolases. c) Transcriptomic analysis of expression changes in extracellular oxidase genes in *A. alternata* FB1 following core MAPK knockout. Genes exhibiting statistically significant differential expressions (*P* < 0.05) were denoted by an asterisk (*).

The dynamic transcriptional changes observed during the interaction between *A. alternata* FB1 and the PU surface suggest that both adhesion and degradation processes are under precise regulatory control. Genomic analysis of strain FB1 identified three MAPKs: Pmk1, Osm1, and Mps1, which are homologous to *Saccharomyces cerevisiae* Fus3/Kss1, Hog1, and Slt2, respectively. Subsequent functional assays showed that the Δ*pmk1* mutant failed to form typical appressoria on PEUR films, whereas the Δ*mps1* mutant produced morphologically aberrant appressoria (Fig 3a). Both mutants exhibited markedly reduced polyurethane-degrading capabilities: after 3 days of incubation, the Δ*pmk1* mutant produced only minor fissures on PAUR films with a substantially diminished oxidized brown zone, and SEM imaging of PEUR films revealed only sporadic micropores; Δ*mps1* caused even fewer cracks and pores, with negligible erosion. Complementation of Δ*pmk1* and Δ*mps1* substantially restored both appressorium formation and PU degradation activity (S5 Fig). These results indicate that the Pmk1/Mps1 MAPK pathway contributes to appressorium differentiation and is associated with efficient polyurethane degradation. Intriguingly, the Δ*osm1* mutant formed normal appressoria on PEUR surfaces, with PAUR fissure numbers and PEUR pore densities comparable to the wild-type strain, but exhibited significantly enlarged oxidized brown regions on both film types, suggesting that Osm1 may negatively regulate oxidative reactions to modulate pigmentation.

Subsequently, melanin biosynthesis genes *pksA*, *brm1*, and *brm2* were deleted to investigate their contribution to appressorium function and polyurethane degradation [27–29]. All melanin-deficient mutants formed appressoria and generated dense fissures on PAUR films but displayed a pale overall appearance with markedly reduced brown oxidized zones (S6 Fig). On PEUR films, degradation patterns further underscored melanin’s importance in penetration structures. Knocking out the polyketide synthase gene *pksA* produced only sparse surface pores, and disruption of the downstream scytalone dehydratase *brm1* moderately reduced pore formation. In contrast, inactivation of the trihydroxy-naphthalene reductase *brm2* restored pore density to wild-type levels but markedly reduced pore depth, resulting in shallow indentations. These findings indicate that melanin biosynthesis is not required for appressorium formation. However, melanin deficiency was associated with impaired penetration-related phenotypes and reduced PEUR degradation.

To uncover the molecular basis of the altered pigmentation phenotypes, RNA-seq analysis was performed on Δ*pmk1*, Δ*mps1*, and Δ*osm1* mutants at 6 h post-PEUR contact, with PEUR-exposed wild-type mycelia serving as the reference condition. The results showed that the Δ*pmk1* and Δ*mps1* strains shared highly similar transcriptional patterns across multiple functional categories, including signal transduction, cellular architecture, cell cycle regulation, and extracellular oxidoreductases/hydrolases (Fig 3b). In both Δ*pmk1* and Δ*mps1* strains, expression of extracellular oxidases was broadly downregulated (Fig 3c), potentially attenuating PU surface oxidation. Concurrently, genes encoding peroxidases and catalases, which mediate reactive oxygen species (ROS) scavenging, were upregulated (S7a Fig), consistent with activation of ROS-detoxification pathways in these mutants. Notably, in Δ*mps1*, expression of the siderophore synthetase Nps6 and other antioxidant transcription factors was significantly enhanced (S7b Fig), potentially reflecting an enhanced oxidative-stress response [30]. In contrast, the Δ*osm1* strain displayed a comparatively divergent transcriptional profile and exhibited upregulated extracellular oxidase expression (Fig 3c), a shift that may contribute to the more pronounced yellowing observed on polymer films. Together, these results support a model in which distinct MAPK pathway branches differentially regulate appressorium-associated development and oxidative processes during polyurethane degradation.

### Appressorium-driven PU degradation pathway in *A. alternata* FB1

**Fig 4:**
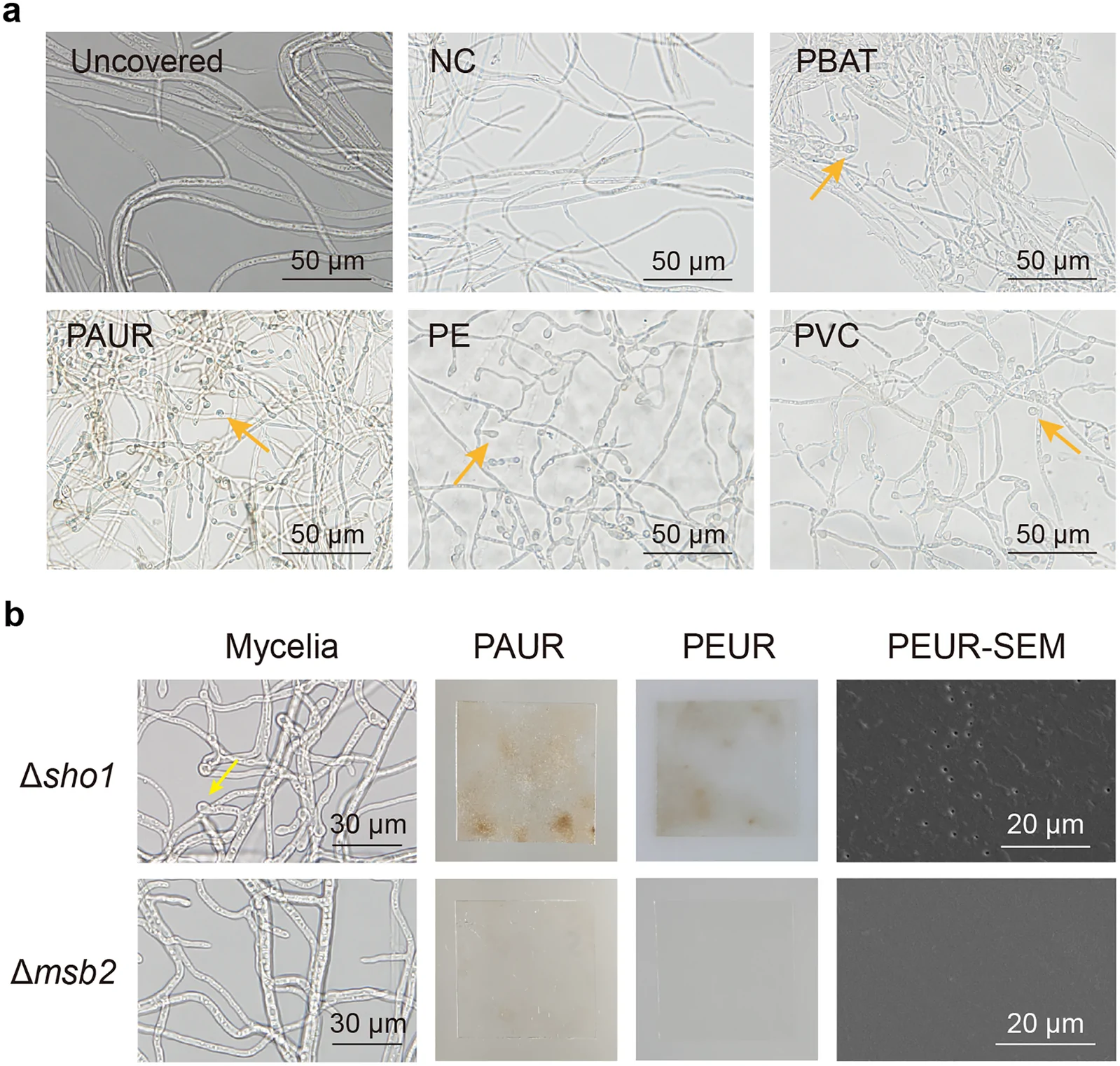
Msb2-mediated hydrophobic sensing is crucial for appressorium formation and polyurethane degradation. a) Hyphal morphology and appressorium formation of *A. alternata* FB1 on substrates of differing wettability. NC, nitrocellulose membrane; PBAT, poly(butylene adipate-*co*-terephthalate); PE, polyethylene; PVC, polyvinyl chloride. b) Effects of deleting putative hydrophobicity receptors on appressorium development and polyurethane-degrading activity of *A. alternata* FB1. “Mycelia” depicts the hyphal morphology of different FB1 knockout strains after 24 h of contact with PU films, with yellow arrows indicating the formation of appressoria structures. “PAUR” and “PEUR” show the macroscopic changes in PAUR or PEUR films, respectively, after three days of co-incubation with each strain. “PEUR-SEM” presents the microstructural morphology of PEUR films observed via SEM after three days of co-incubation with each strain.

Building upon the insights gleaned above, the signaling cascade that couples MAPK‑dependent metabolic reprogramming to the physical onset of polyurethane erosion remains to be fully elucidated. To determine whether surface hydrophobicity serves as a cue for appressorium differentiation, films with different surface properties were co-incubated with wild-type mycelia for 24 h (Fig 4a). Mycelia in contact with hydrophilic surfaces exhibited morphology indistinguishable from non‑contact controls and did not form appressoria. In contrast, contact with all hydrophobic matrices induced swollen appressorium structures, indicating that substrate hydrophobicity is sufficient to induce appressorium differentiation.

Given that hydrophobic sensing may serve as a key signal for appressorium differentiation, we identified and deleted homologs of the candidate sensor proteins Sho1 and Msb2 [31]. Sho1 comprises four transmembrane helices and a cytoplasmic SH3 domain, whereas Msb2 is a single-pass, heavily glycosylated signaling mucin. As previously reported, both sensors converge on MAPK modules to detect osmotic stress and nutrient scarcity, cooperating physically and functionally to activate downstream pathways [32,33]. In the Δ*sho1* mutant, appressorium formation and polyurethane-degrading activity remained comparable to those of the wild type (Fig 4b). However, the Δ*msb2* strain failed to form appressoria on PEUR, produced only minimal fissures on PAUR films, and showed no pore formation on PEUR surfaces (Fig 4b). These observations indicate that Msb2, independent of the canonical Sho1 sensor, plays a central role in hydrophobic surface sensing and is required for efficient appressorium differentiation and polyurethane degradation in *A. alternata* FB1.

Transcriptomic analysis comparing PEUR-exposed Δ*msb2* and wild-type mycelia further helped distinguish primary surface-sensing defects from downstream adaptive responses. The Δ*msb2* mutant showed significant downregulation of extracellular oxidase genes and multiple translation factor genes (S8 Fig), consistent with impaired activation of PU-associated degradative and biosynthetic programs following defective hydrophobic sensing. In contrast, the upregulation of autophagy‑related genes is more likely to represent a secondary stress-adaptation response, possibly reflecting compensatory recycling of cellular components or clearance of damaged organelles when normal surface recognition and appressorium development are disrupted.

Comparative transcriptomic profiling of the Δ*pmk1*, Δ*mps1*, and Δ*msb2* mutants, which each exhibit similar impairments in appressorium formation and degradation, uncovered a cohort of commonly downregulated genes likely involved in appressorium development and polyurethane degradation. This shared transcriptional signature suggests the existence of a coordinated regulatory network underlying adhesion structure differentiation and substrate utilization.

**Fig 5:**
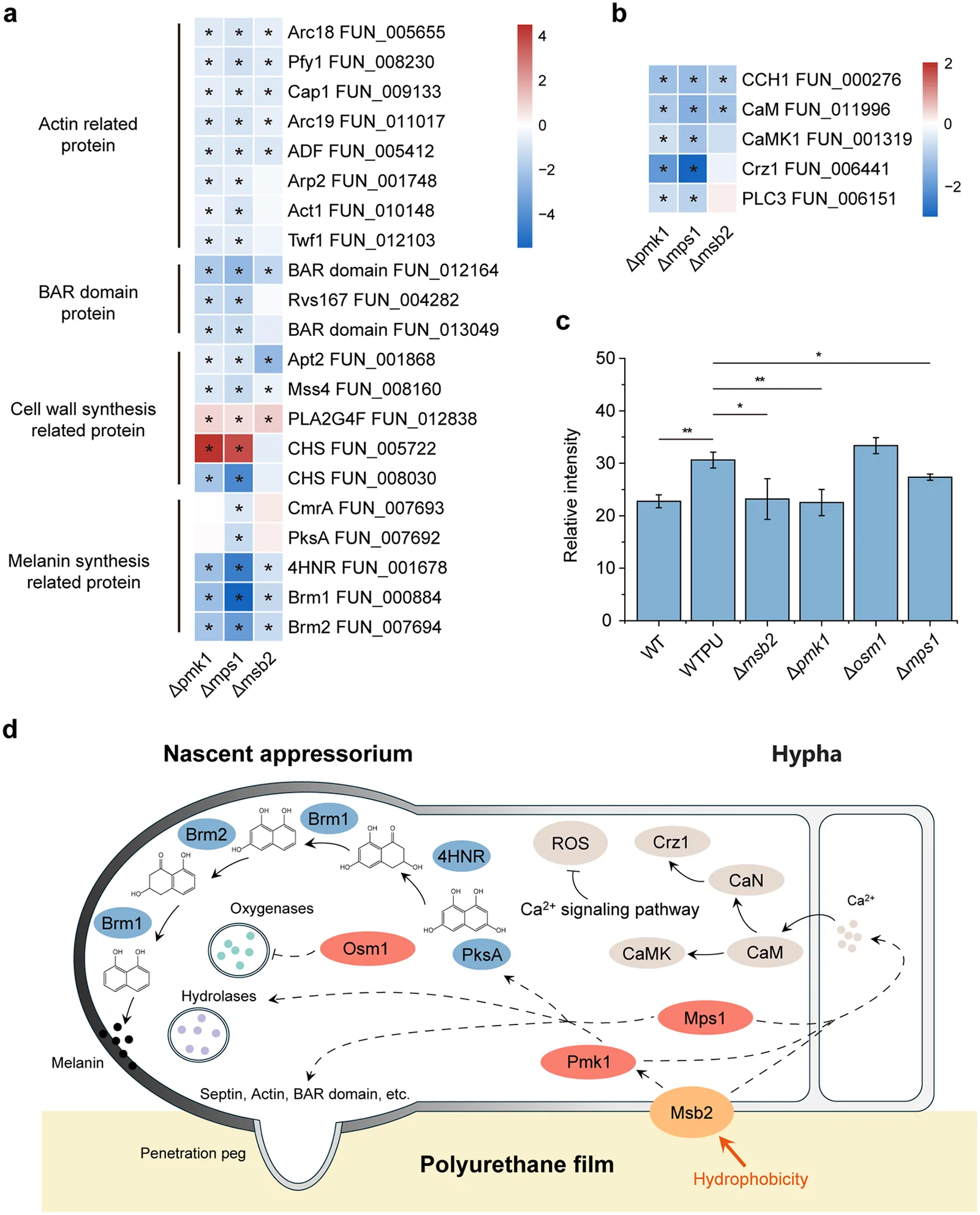
Coordinated transcriptional responses and mechanistic model of appressorium-driven PU degradation. a) Appressorium formation-associated structural components and cell wall integrity–related genes with concordant expression changes across Δ*pmk1*, Δ*mps1*, and Δ*msb2*. b) Ca^2+^ signaling pathway-related genes exhibited similar expression alterations across Δ*pmk1*, Δ*mps1*, and Δ*msb2*. Genes exhibiting statistically significant differential expressions (*P* < 0.05) were denoted by an asterisk (*). c) Relative intracellular Ca^2+^ concentrations in wild-type and knockout strains following contact with polyurethane films. “WT” denotes wild-type strain unexposed to the polyurethane film, whereas “WTPU” refers to wild-type strain following contact with the film. “Δ*msb2*”, “Δ*pmk1*”, “Δ*osm1*”, and “Δ*mps1*” correspond to the respective gene‐knockout strains after film exposure. All measurements were conducted in triplicate and shown as mean ± s.d. (n = 3); *P < 0.05, **P < 0.01 (two-sided *t*-test). d) Schema*t*ic model illustrating appressorium-mediated polyurethane degradation by *A. alternata* FB1, highlighting hydrophobic signal perception, MAPK cascade activation, melanin biosynthesis, cytoskeletal remodeling, and secretion of degradative enzymes. The abbreviations in the diagram are as follows: Msb2, the mucin‐like sensor; Pmk1, Mps1, and Osm1, core mitogen-activated protein kinases (MAPKs); PksA, polyketide synthase; 4HNR, tetrahydroxynaphthalene reductase; Brm1, scytalone dehydratase; Brm2, trihydroxynaphthalene reductase; CaM, calmodulin; CaN, calcineurin; CaMK, Ca^2+^/calmodulin-dependent protein kinase; Crz1, calcineurin-responsive transcription factor.

Among the repressed genes were those encoding the phospholipid flippase Apt, phosphatidylinositol kinase Mss4, BAR-domain proteins, and multiple actin-associated factors (Fig 5a). These components are functionally linked to adhesion structure differentiation, membrane dynamics regulation, and the maintenance of cellular mechanical strength. In addition, Ca^2+^ signaling pathway-related genes were consistently downregulated in the three mutants (Fig 5b), indicating potential disruption of calcium-dependent regulatory processes. To validate this transcriptomic pattern at the physiological level, intracellular Ca^2+^ dynamics were quantified using fluorescence-based assays following 6 h of contact with polyurethane films. The wild-type strain exhibited a pronounced Ca^2+^ influx under these conditions, whereas Δ*msb2*, Δ*pmk1*, and Δ*mps1* failed to elevate cytosolic Ca^2+^ to comparable levels, underscoring the specific requirement for Ca^2+^ mobilization during early appressorium induction (Fig 5c). In parallel, transcription of peptidase-encoding genes was markedly decreased across these mutants, while transcripts encoding lipases and esterases were concomitantly up-regulated (S9 Fig). This pattern suggests that peptidases are likely important contributors to polyurethane depolymerization and that their diminished expression may compromise degradation efficiency.

In summary, our findings delineate a coordinated signaling network that governs *A. alternata* FB1’s recognition of polyurethane surfaces, appressorium differentiation and polymer degradation (Figs 5d, S10). The mucin-like sensor Msb2 is proposed to function in hydrophobic surface sensing and to act upstream of the MAPK Pmk1. In concert with Mps1, Pmk1 orchestrates melanin biosynthesis, cytoskeletal reorganization, and Ca^2+^ signaling to promote appressorium maturation and mechanical penetration of the polymer matrix. Simultaneously, this MAPK module is associated with increased expression of cutinases, peptidases, and oxidases, which are likely to contribute to oxidative and hydrolytic depolymerization of PU. The osmotic-stress‐responsive MAPK Osm1 fine-tunes oxidative degradation by repressing extracellular oxidase expression. The distinct expression profiles of these signaling components and effectors underlie the phenotypic defects observed in their respective deletion mutants, thereby providing novel mechanistic insights into fungal appressorium formation and polyurethane biodegradation.

## Discussion

Plastic biodegradation is a complex, multistage process encompassing surface adhesion, substrate recognition, enzymatic depolymerization, and ultimate assimilation. To deepen understanding of this process, we evaluated the polyurethane-degrading capability of the marine fungus *A. alternata* FB1 and elucidated the molecular mechanisms underlying appressorium-mediated degradation. The results demonstrate that *A. alternata* FB1 employs diversified degradation strategies tailored to different polyurethane types. For PAUR, degradation is driven primarily by synergistic hydrolysis and mechanical penetration, aligning with the loss of ester-bond signals observed in fungal-treated films and previous reports for *Aspergillus flavus* [19]. In contrast, degradation of PEUR by *A. alternata* FB1 predominantly relies on oxidative scission targeting the polymer’s soft segments, leading to altered thermal properties and molecular-weight distribution, a pattern also reported for other hydrolysis-resistant polymers [34,35].

Together with the early transcriptomic and microscopic observations, these findings support a temporal model in which hydrophobic surface sensing initiates appressorium differentiation, pore formation, and activation of degradative pathways, ultimately driving polyurethane deterioration during prolonged incubation. *A. alternata* FB1 generates dense surface pores on polyurethane films within 24 h, a response that appears considerably faster than those reported for most polyurethane-degrading microorganisms. The rapid formation of these micropores likely represents a critical initiating step, reflecting appressorium-mediated penetration while simultaneously increasing the accessibility of the polymer matrix to extracellular degradative enzymes. By disrupting surface integrity and exposing internal polymer chains, such localized perforation may facilitate subsequent oxidative and hydrolytic reactions, thereby linking early appressorium activity to the extensive physicochemical changes observed at later stages of degradation.

Our results confirm that this rapid response reflects an active surface-recognition process that initiates PU degradation by strain FB1, rather than a nonspecific stress response alone. The Msb2-Pmk1 MAPK cascade integrates early surface sensing with Ca^2+^ homeostasis during appressorium morphogenesis. As a transmembrane sensor, Msb2 integrates external physical cues to activate the downstream MAPK cascade [31], and the MAPK Pmk1 serves as the core effector, directly contributing to appressorium maturation and penetration force generation [36]. During appressorium development, Ca^2+^ serves as a second messenger that regulates cytoskeletal dynamics, transmembrane signal transduction, and mechanical penetration capability [24,25]. Weakened calcium signaling may impair fungal responsiveness to environmental stimuli. However, genes associated with the cAMP-PKA pathway exhibited no significant changes in the mutants. Given this pathway’s synergistic role with MAPK cascades in appressorium regulation in *M. oryzae* [22], further investigation of intracellular cAMP levels in *A. alternata* FB1 post-PU contact is warranted to elucidate its regulatory contributions.

Upon perception of the PU surface, membrane-remodeling proteins and cytoskeletal components appear to convert external physical cues into mechanically competent penetration structures. Genetic perturbations of the Msb2-MAPK module provide direct support for this interpretation. Deletion of the MAPK kinases Pmk1 or Mps1, or of the signaling mucin Msb2, simultaneously (i) abolishes polyurethane erosion, (ii) distorts appressorium morphology, and (iii) suppresses transcripts encoding the Apt2 and Mss4, enzymes that sustain membrane fluidity and structural integrity [22]. Concomitant downregulation of BAR-domain proteins, which sense and remodel membrane curvature, is likely to further disrupt penetration peg formation [37]. These concerted transcriptional changes pinpoint membrane remodeling as an important downstream effector of MAPK signaling. Conversely, the uniform upregulation of cytosolic phospholipase A_2_ (PLA2G4F) across all mutants (Fig 5a) may reflect a compensatory attempt associated with membrane stress and cellular adaptation after disruption of the primary recognition pathway [38]. Overall, these phenotypic and transcriptomic signatures converge to demonstrate that Msb2-MAPK-dependent signaling organizes the early membrane and cytoskeletal machinery required for attachment, penetration and subsequent polyurethane degradation by *A. alternata* FB1.

Although the strong correlation between impaired appressorium formation and reduced polyurethane degradation supports a functional role for appressoria in polymer penetration, alternative explanations should also be considered. Pmk1, Mps1, and Msb2 regulate multiple aspects of fungal physiology beyond appressorium development, including cell-wall integrity, stress adaptation, protein secretion, and developmental processes. Therefore, the reduced degradation observed in the corresponding mutants may partly reflect broader physiological defects that indirectly influence polymer colonization and enzyme delivery.

In addition to regulating appressorium development and membrane remodeling, MAPK signaling also influences melanin biosynthesis and extracellular oxidative responses. Melanin is likely to strengthen appressorial cell walls and support the generation of penetration force, thereby facilitating physical entry into the polymer matrix [39]. Consistently, melanin-deficient mutants formed appressoria but showed reduced PEUR pore formation or shallower surface damage, indicating that melanin contributes mainly to the mechanical competence of penetration structures rather than to their initial formation. At the same time, MAPK mutants displayed altered expression of extracellular oxidases and ROS-related genes, suggesting that oxidative modification of PU surfaces is also under MAPK-associated regulation. However, these effects may reflect both direct regulatory outputs and broader physiological changes in the mutants. Subsequent depolymerization is likely mediated by hydrolytic enzymes, as supported by the detection of degradation products such as butanediol and MDA and by the induction of peptidase-related genes. Together, these observations support a model in which appressorium-mediated penetration, oxidative surface modification, and enzymatic hydrolysis act cooperatively during polyurethane degradation by A. alternata FB1.

The findings of this study raise the possibility that appressorium-mediated interactions with synthetic materials may extend beyond *A. alternata* FB1. Phytopathogenic *Fusarium* species elaborate lobate and compound appressoria to breach plant tissues under tightly regulated developmental cues [40] and have been shown to mineralize recalcitrant pollutants, including synthetic plastics [41,42]. Likewise, biocontrol *Trichoderma* strains deploy appressorium‐like structures to invade pathogenic hyphae and exhibit an activity in which laccases play a pivotal role in polyethylene depolymerization [43,44]. Plant pathogens such as *Colletotrichum* and *Botrytis*, and insect pathogens like *Beauveria*, all form specialized penetration organs that may harbor unique suites of oxidative and hydrolytic enzymes [45–47]. Comparative investigation of these fungi across different polymer classes, including polyester, polyether, and polyolefin, may help identify conserved and lineage-specific mechanisms linking surface sensing, penetration, and polymer degradation. More broadly, integrating concepts from fungal infection biology with studies of polymer biodegradation may provide new opportunities to understand how microorganisms interact with synthetic materials and to develop improved strategies for biological plastic conversion.

## Materials and methods

### Chemicals

Polyester and polyether polyurethane films were obtained from Guangzhou HuanYou Polymer New Materials Co., Ltd. in China, with nominal thicknesses of 0.2 mm and 0.3 mm, respectively. The PAUR films were formulated from 4,4’-methylenebis(phenyl isocyanate) (MDI), adipic acid (AA), 1,4-butanediol (BDO), and 1,6-hexanediol (HDO) (S11 Fig). The PEUR films were synthesized from MDI, poly(tetramethylene ether) glycol (PTMG), and BDO (S12 Fig).

### Fungal strains, growth conditions, and assessment of PU degradation

The *A. alternata* strain FB1 used in this study was previously isolated from the intertidal locations in the Huiquan Bay (Qingdao, China) [14]. To generate sufficient biomass for downstream assays, mycelia of *A. alternata* FB1 (wild-type or gene-knockout strains) were transferred into basal liquid medium (0.05 g yeast extract and 0.02 g xylose per L of filtered seawater) and incubated at 28 °C and 160 rpm for 2 ~ 3 days. The appearance of white, fluffy mycelial balls in the culture served as the seed inoculum.

PAUR and PEUR films were cut into 2 × 2 cm squares, rinsed thoroughly, and immersed in 75% (v/v) ethanol for 2 h. Prior to use, the films were retrieved in a laminar‐flow hood, washed with sterile deionized water, air-dried, and then exposed to UV irradiation for 15 min to ensure sterility. Activated mycelial seeds were aseptically inoculated onto rice‐solid medium (0.1 g corn flour, 0.3 g tryptone, 0.5 g yeast extract, 0.2 g monosodium glutamate, and 50 g rice per 100 mL of filtered seawater) and incubated statically at 28 °C. After 3 ~ 4 days, a sterile PAUR or PEUR film square was gently overlaid onto the fungal mat. At the designated time point, films co-cultured with *A. alternata* FB1 were retrieved and soaked overnight in 1% (v/v) hydrogen peroxide to detach the mycelium. Films were then sonicated for 15 min to remove surface-adhered biomass. For PAUR films exhibiting strong mycelial adhesion, the peroxide-soak and sonication steps were repeated 2 ~ 3 times. Treated films were air-dried and subsequently analyzed to quantify the extent of fungal degradation of PAUR and PEUR.

### Scanning electron microscopy (SEM) observation

To examine the colonization of *A. alternata* FB1 on the polyurethane film, samples were prepared as follows. At the designated incubation time, PU films bearing fungal hyphae were gently rinsed with 10 mM sterile phosphate-buffered saline (PBS) to remove loosely adhered material. Specimens were then fixed in 5% (v/v) glutaraldehyde for 30 ~ 60 min, followed by thorough washing in PBS to remove residual fixative. Dehydration was performed through a graded ethanol series (10%, 30%, 50%, 70%, 90%, and 100%), with each step lasting 10 min. After the final ethanol wash, samples were air-dried. For visualization of fungal colonization, dried samples were mounted onto aluminum stubs and sputter-coated with a 10 nm layer of platinum using an MC1000 ion sputter coater (Hitachi, Japan). To observe the underlying PU surface morphology post-colonization, a parallel set of samples was treated to remove surface hyphae, air-dried, and then gold-coated. All specimens were examined using an S-3400N (Hitachi, Japan) operated at an accelerating voltage of 5 kV.

### Transcriptome sequencing and analysis

Activated *A. alternata* FB1 seed cultures were grown on rice‐solid medium at 28 °C for 3 ~ 4 days. Then a sterile PAUR or PEUR film square was gently overlaid onto the fungal mat, and this moment was defined as time zero. Experimental samples were harvested at 6 h and 24 h by carefully excising the mycelium in direct contact with the film; simultaneously, surface mycelium from adjacent regions not covered by the film was collected as the control. For gene‐knockout strains, mycelium from wild‐type cultures contacting the film served as the control group, while film‐contacting knockout mycelium constituted the experimental group. Each condition was performed in biological triplicate.

Harvested mycelial samples were immediately flash‐frozen in liquid nitrogen, stored on dry ice, and shipped to Beijing Novogene Bioinformatics Technology Co., Ltd. for RNA extraction and high‐throughput sequencing. In brief, extracted RNA integrity was first assessed using an Agilent 2100 Bioanalyzer, and the resulting libraries were subjected to high‑throughput sequencing on Illumina NovaSeq X Plus. After removal of adaptor sequences and low-quality reads, clean paired-end reads were mapped to the *A. alternata* FB1 reference genome [14] using HISAT2 v2.0.5 with default parameters. Differential expression analysis between experimental and control groups was performed using the DESeq2 R package v1.20.0. *P* values were adjusted using the Benjamini-Hochberg method to control the false discovery rate.

### Effect of membrane hydrophobicity on appressorium formation

Polyethylene (PE), polyvinyl chloride (PVC), PAUR, PEUR, and nitrocellulose membranes were cut into 2 × 2 cm squares, rinsed thoroughly, and sterilized by immersion in 75% (v/v) ethanol for 2 h. Before use, membranes were transferred under a laminar‐flow hood, washed with sterile deionized water, air‐dried, and then exposed to UV irradiation for 15 min to ensure sterility.

*A. alternata* FB1 seed cultures were produced on V8 agar (200 mL V8 juice, 3 g CaCO_3_, and 15 g agar per L of distilled water) at 28 °C for 3 ~ 4 days. Sterile membrane squares were gently overlaid on the resulting fungal mat. After 24 h of static incubation at 28 °C, membranes were removed and immediately examined under a light microscope to assess appressorium formation on each substrate.

### Ultrathin sectioning and transmission electron microscopy (TEM) observation

To investigate the interface between *A. alternata* FB1 hyphae and polyurethane films, ultrathin sections were prepared for TEM. A sterile PU film was placed onto rice‐solid medium colonized by wild-type FB1 and incubated statically at 28 °C for 24 h. Films were then rinsed in 10 mM sterile PBS to remove loosely attached material and fixed in 2.5% (v/v) glutaraldehyde. Fixed samples were sent to Qingdao University, where they were embedded in Epon-812 resin and sectioned using an ultramicrotome. Sections were examined on a HT7700 (Hitachi, Japan) to visualize hyphal penetration and film deformation.

### 3,3’-diaminobenzidine (DAB) staining

PU films bearing attached fungal hyphae were vacuum-infiltrated in DAB staining solution to detect hydrogen peroxide accumulation. Samples were incubated in the dark at room temperature for 2 h, then fixed overnight in an ethanol: acetic acid mixture (96:4, v/v) [17]. DAB-stained specimens were rinsed, air-dried, and observed by light microscopy to localize oxidative activity associated with appressorium formation.

### Generation of *A. alternata* FB1 gene-knockout mutants

The construction of FB1 gene knockout strains was performed as previously described [48]. In brief, upstream and downstream flanking regions of the *pmk1*, *osm1*, *mps1*, *pksA*, *brm1*, *brm2*, *sho1*, and *msb2* genes were amplified using primers listed in Table A in S1 Appendix. An overlap-extension PCR strategy was then employed to fuse the upstream homology arm, the promoter-driven hygromycin-B phosphotransferase (*hph*) resistance cassette, and the downstream homology arm into a single deletion construct. Recombinant fragments were introduced into wild-type *A. alternata* FB1 protoplasts via polyethylene glycol-mediated transformation. Transformants were selected on potato dextrose agar (6 g potato extract, 20 g glucose, 20 g agar per L of distilled water) supplemented with 100 μg/mL hygromycin B and subcultured five times to ensure stability. Genomic DNA was extracted using a fungal genomic extraction kit (Solarbio, China), and successful gene replacements were confirmed by PCR amplification and Sanger sequencing using the external primer pairs detailed in Table A in S1 Appendix.

## Supporting information

S1 Appendix Supplementary methods and tables. (DOCX)

S1 Table Relative intracellular Ca^2+^ levels after polyurethane contact. (XLSX)

S1 Rawgel Original uncropped gel images corresponding to S5 Fig. (PDF)

S1 Fig Thermal transition characteristics of PEUR films before and after 28 days of *A. alternata* FB1 treatment analyzed by differential scanning calorimetry (DSC). (TIF)

S2 Fig Potential degradation products after the treatment of polyurethane by *A. alternata* FB1. After 28 days of co-incubation with *A. alternata* FB1, the potential degradation products were analyzed and classified into three categories: “PAUR & PEUR” refers to degradation products detected in both PAUR and PEUR samples, while “PAUR” and “PEUR” indicate products detected exclusively in PAUR or PEUR samples, respectively.
(TIF)

S3 Fig Transcriptomic analysis reveals genes potentially involved in *A. alternata* FB1-mediated polyurethane degradation. Transcriptomic analysis was performed after *A. alternata* FB1 was co-incubated with polyurethane films for 24 hours. The results indicated significant changes in gene expression (*P* < 0.05) upon contact with PU films, including ester bond hydrolase genes (a), amide bond hydrolase genes (b), and extracellular oxidase genes (c). Gene expression changes are presented as log_2_-transformed fold changes. Each experimental group included three independent biological replicates.
(TIF)

S4 Fig Transcriptomic analysis reveals gene expression changes related to cell cycle (a) and DNA replication (b) in *A. alternata* FB1 during incubation with PEUR film. Differentially expressed genes were identified based on a significance threshold of *P* < 0.05 following 6 h and 24 h of co-incubation with the film.
(TIF)

S5 Fig Genetic complementation restores appressorium formation and polyurethane degradation in the Δ*pmk1* and Δ*mps1* mutants. a) Molecular confirmation of the complemented strains. “WT” represents the wild-type *A. alternata* FB1. b) Colony morphology of the wild-type, knockout mutant, and complemented strains. c) Restoration of appressorium formation and polyurethane degradation on PU films. “Mycelia” depicts the hyphal morphology of different FB1 complemented strains after 24 h of contact with PU films, with yellow arrows indicating the formation of appressoria structures. “PAUR” and “PEUR” show the macroscopic changes in PAUR or PEUR films, respectively, after three days of co-incubation with each strain. “PEUR-SEM” presents the microstructural morphology of PEUR films observed via SEM after three days of co-incubation with each strain.
(TIF)

S6 Fig Impact of deletion of core melanin biosynthesis genes on appressorium foration and the polyurethane degradation ability of *A. alternata* FB1. “WT” represents the wild-type *A. alternata* FB1, serving as the control. “Mycelia” depicts the hyphal morphology of different FB1 knockout strains after 24 h of contact with PU films, with yellow arrows indicating the formation of appressoria structures. “PAUR” and “PEUR” show the macroscopic changes in PAUR or PEUR films, respectively, after three days of co-incubation with each strain. “PEUR-SEM” presents the microstructural morphology of PEUR films observed via SEM after three days of co-incubation with each strain.
(TIF)

S7 Fig Expression changes in ROS detoxification-related genes in *A. alternata* FB1 following core MAPK kinase knockout. a) Differential expression of ROS detoxification-related genes in Δ*pmk1* and Δ*mps1* mutants. b) ROS detoxification-related genes significantly altered exclusively in the Δ*mps1* mutant but not in Δ*pmk1*.
(TIF)

S8 Fig Changes in the transcriptional levels of *A. alternata* FB1 extracellular oxidase genes (a), autophagy-related genes (b), and translation factors (c) after knockout of *msb2.* (TIF)

S9 Fig Hydrolase-encoding genes significantly differentially expressed in Δ*pmk1*, Δ*mps1*, and Δ*msb2.* (TIF)

S10 Fig Schematic timeline of the experimental sequence and the corresponding biological processes. (TIF)

S11 Fig Composition identification of polyester polyurethane films. a) FTIR spectrum of the polyester polyurethane (PAUR) film. b) ^1^H NMR spectrum of the PAUR film. The chemical shifts around 9.54 ppm, 7.35 ppm, 7.08 ppm, and 3.78 ppm correspond to the hydrogen protons on 4,4’-diphenylmethane diisocyanate (MDI). The chemical shift around 2.30 ppm corresponds to the hydrogen protons on adipic acid. c) The thermal pyrolysis products of PAUR films were analyzed using Py-GCMS. The primary detected pyrolysis products included MDI, 1,4-butanediol, 1,6-hexanediol, and 1,6-dioxacyclododecane-7,12-dione.
(TIF)

S12 Fig Composition identification of polyether polyurethane films. a) FTIR spectrum of the polyether polyurethane (PEUR) film. b) The thermal pyrolysis products of PEUR films were analyzed using Py-GCMS. The primary detected pyrolysis products included MDI, 1,4-butanediol, and cyclic fragments of polytetramethylene glycol.
(TIF)
