# Supplementary material for: Fungal appressoria as mechanochemical organelles for polyurethane degradation
Source: PLoS Genet. 2026 Aug 31;22(8):e1012292. doi: 10.1371/journal.pgen.1012292 (PMC13552942; doi:10.1371/journal.pgen.1012292)
Supplement: Supplementary file 1 [file pgen.1012292.s003.pdf]

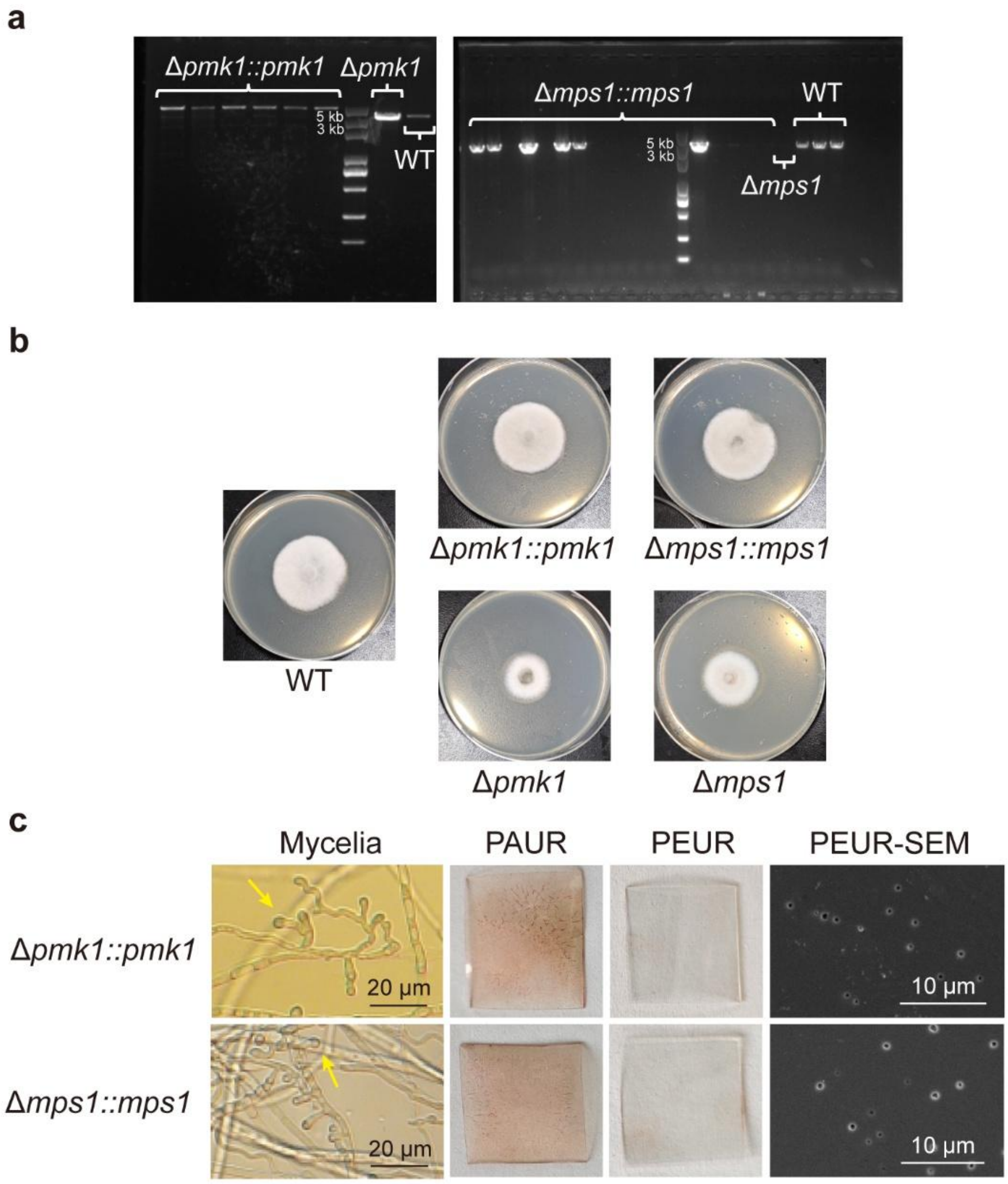

Fig. S5. Genetic complementation restores appressorium formation and polyurethane degradation in the Δ*pmk1* and Δ*mps1* mutants.

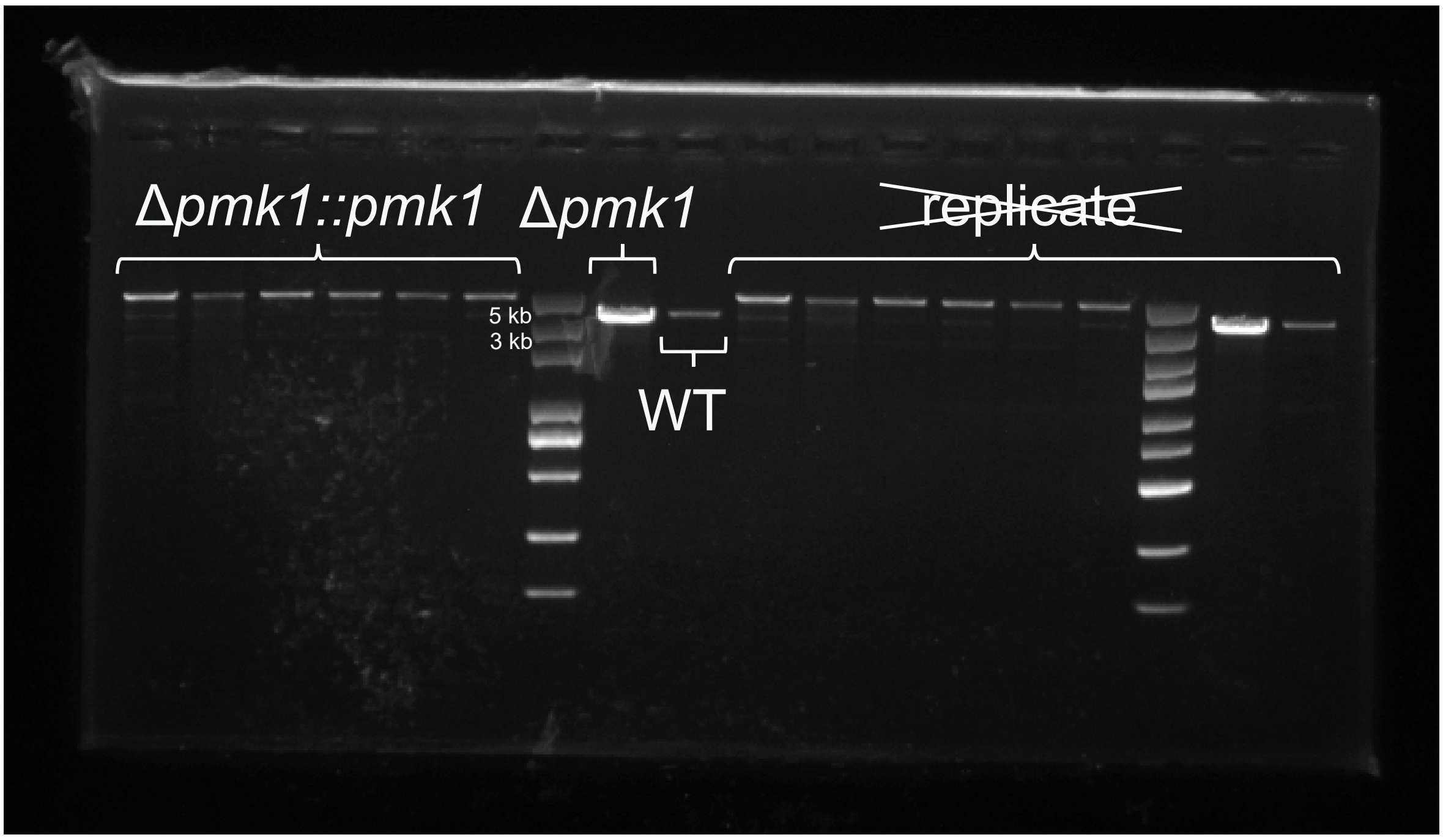
Δpmk1::pmk1
Δpmk1
replicate
5 kb
3 kb
WT

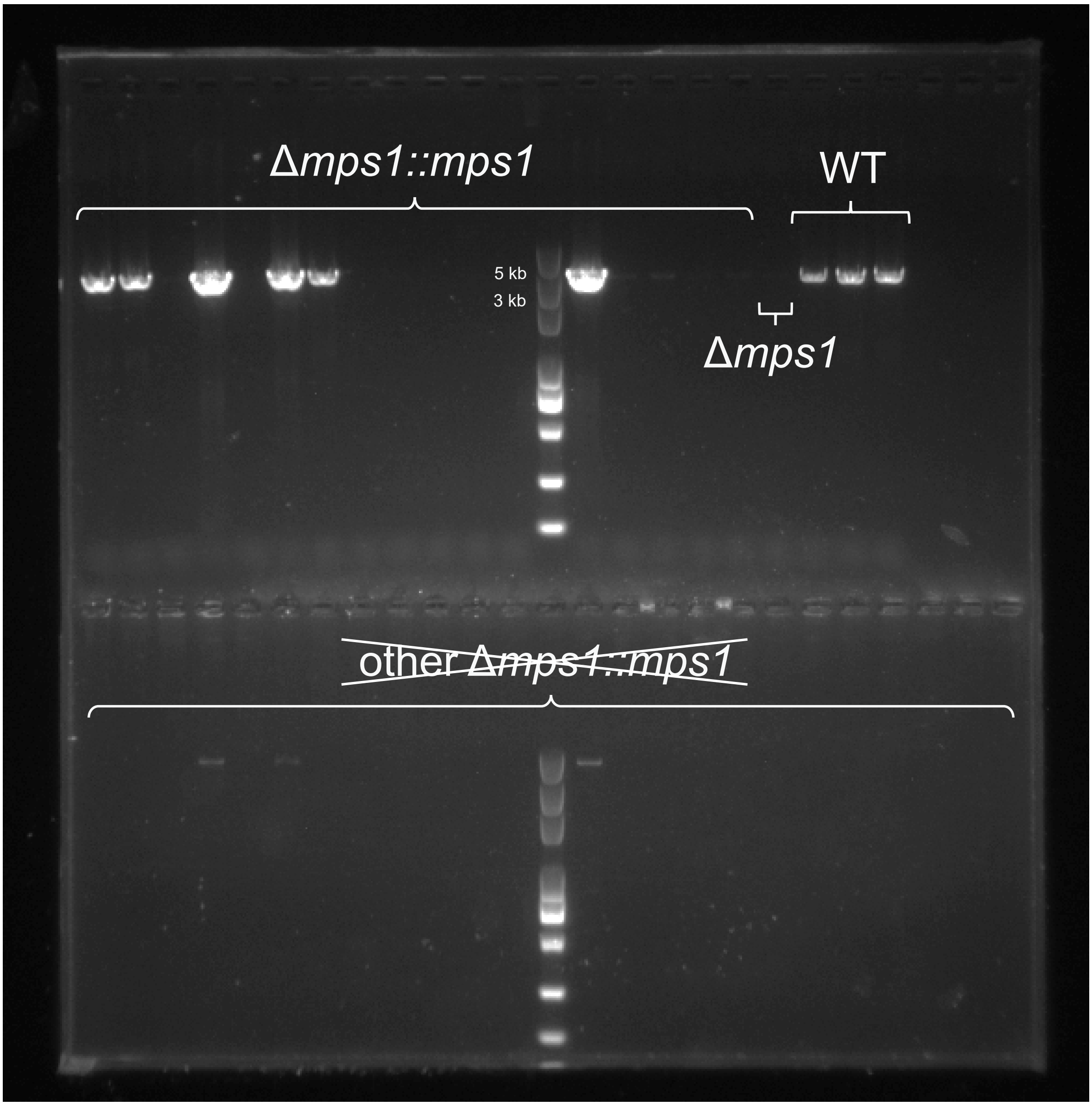
Δmps1::mps1
WT
5 kb
3 kb
Δmps1
other Δmps1::mps1
