# Supplementary material for: Fungal appressoria as mechanochemical organelles for polyurethane degradation
Source: PLoS Genet. 2026 Aug 31;22(8):e1012292. doi: 10.1371/journal.pgen.1012292 (PMC13552942; doi:10.1371/journal.pgen.1012292)
Supplement: Supplementary file 2 [file pgen.1012292.s017.pdf]

Responses to the Editor and Reviewer

Editor's comments

Comment 1: The RNAseq analyses need to be explained in more details both in the results section and in the methods. E.g. the methods do not mention mapping parameters, DESeq2 steps, or the actual reference used. It is not clear which Biosample ID corresponds to which treatment group and replicate. Please extend.

Reponse: Thank you for this helpful suggestion. We have expanded the RNA-seq methods to include the reference genome used, read-mapping procedure, and DESeq2-based differential expression analysis. To improve data transparency, we have added a new Supplementary Table (Table S2) listing all BioSample accession numbers together with their corresponding treatment groups and biological replicates. In addition, we have clarified the experimental design and comparison strategy in the Results section. The revised text explicitly describe the control and treatment groups, biological replicates, and the reference conditions used for each transcriptomic comparison.

The following text has been added to the Methods section:

"After removal of adaptor sequences and low-quality reads, clean paired-end reads were mapped to the *A. alternata* FB1 reference genome using HISAT2 v2.0.5 with default parameters. Differential expression analysis between experimental and control groups was performed using the DESeq2 R package v1.20.0. *P* values were adjusted using the Benjamini-Hochberg method to control the false discovery rate." (Page 19)

"Detailed BioSample accession numbers and their corresponding treatment groups are provided in Table S2." (Page 20)

Table S2. BioSample accession numbers and corresponding experimental groups used for RNA-seq analysis.

Experimental group	Replicate	BioSample ID
Control (6 h)	R1	SAMN49496933
Control (6 h)	R2	SAMN49496934
Control (6 h)	R3	SAMN49496935
PEUR-treated (6 h)	R1	SAMN49496936
PEUR-treated (6 h)	R2	SAMN49496937
PEUR-treated (6 h)	R3	SAMN49496938
Control (24 h)	R1	SAMN49496939
Control (24 h)	R2	SAMN49496940
Control (24 h)	R3	SAMN49496941
PEUR-treated (24 h)	R1	SAMN49496942
PEUR-treated (24 h)	R2	SAMN49496943
PEUR-treated (24 h)	R3	SAMN49496944
Δ*pmk1* + PEUR (6 h)	R1	SAMN49496945
Δ*pmk1* + PEUR (6 h)	R2	SAMN49496946
Δ*pmk1* + PEUR (6 h)	R3	SAMN49496947
Δ*osm1* + PEUR (6 h)	R1	SAMN49496948
Δ*osm1* + PEUR (6 h)	R2	SAMN49496949
Δ*osm1* + PEUR (6 h)	R3	SAMN49496950
Δ*mps1* + PEUR (6 h)	R1	SAMN49496951
Δ*mps1* + PEUR (6 h)	R2	SAMN49496952
Δ*mps1* + PEUR (6 h)	R3	SAMN49496953
Δ*msb2* + PEUR (6 h)	R1	SAMN49496954
Δ*msb2* + PEUR (6 h)	R2	SAMN49496955
Δ*msb2* + PEUR (6 h)	R3	SAMN49496956

The following text has been added to the Results section:

"To identify genes involved in polyurethane degradation, RNA-seq was performed on mycelia directly contacting PU films for 24 h, using adjacent surface mycelia not covered by the films as controls (three biological replicates per group). Comparative transcriptomic analysis revealed a coordinated enzymatic response underpinning polyurethane degradation." (Page 7)

"Because appressoria form rapidly upon contact with solid surfaces, RNA-seq analysis was performed after 6 h of PEUR exposure, using adjacent non-contact mycelia as controls, to distinguish early PU recognition from later adaptive responses." (Page 8)

Comment 2: I agree with the reviewer that the lack of complementation of mutants is a key drawback of this manuscript and must be either addressed with additional experiments or a more nuanced discussion. In addition, more pleitrophic effects of these mutants must be considered as well.

Response: Thank you for this valuable comment. We fully agree that additional genetic validation would strengthen the conclusions of this study. In response, we performed complementation analyses for the Δ*pmk1* and Δ*mps1* mutants. Restoration of the corresponding wild-type genes rescued both appressorium formation and polyurethane degradation, providing independent genetic evidence that the observed phenotypes are attributable to loss of PmkK1 and Mps1. These complementation data have been incorporated into the revised manuscript (Fig. S5) and further reinforce the proposed role of the MAPK pathway in polyurethane degradation. Although we also attempted to complement Δ*msb2*, a stable complemented strain has not yet been obtained. We therefore revised the manuscript to present the conclusions regarding Msb2 more cautiously.

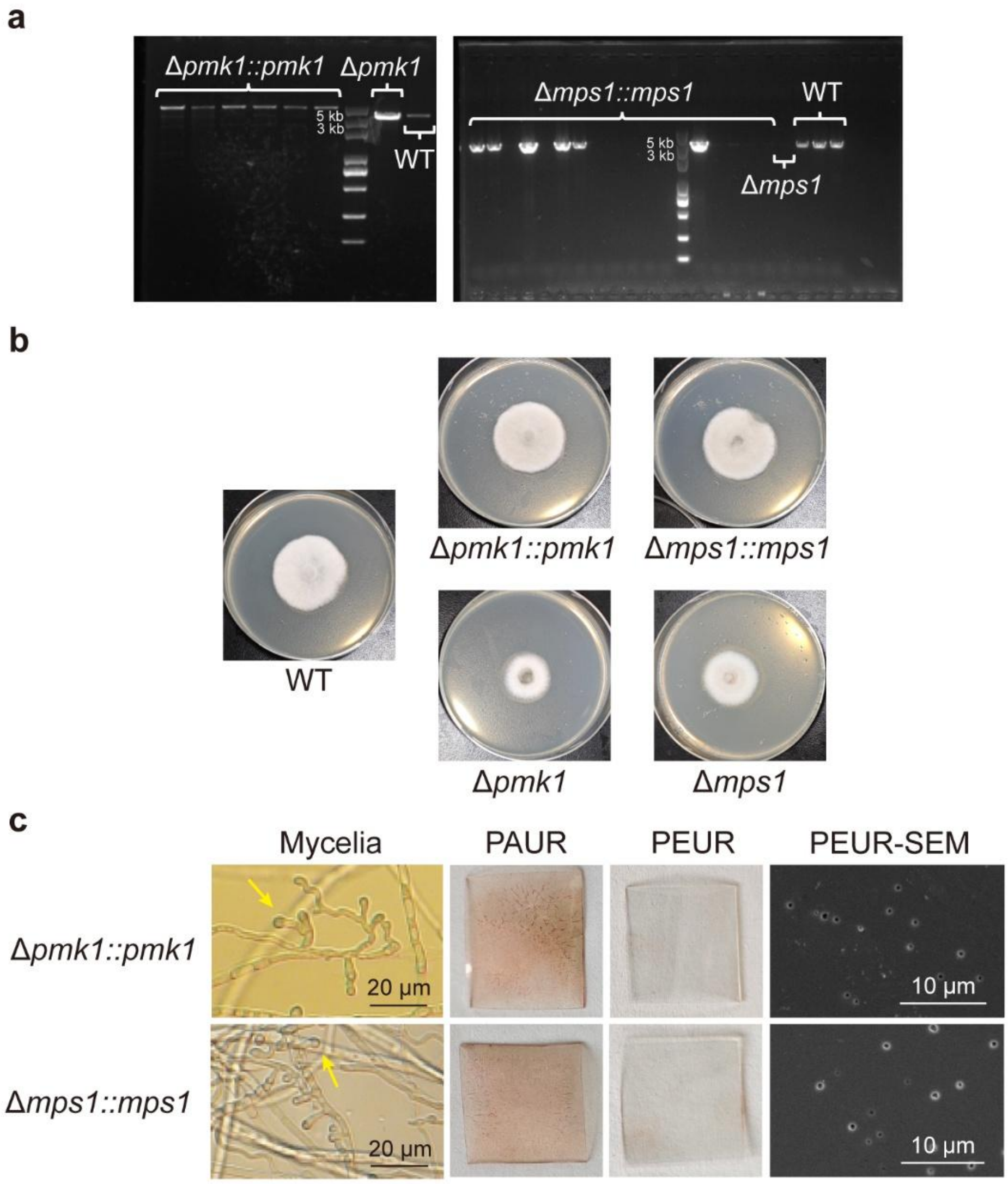

Fig. S5. Genetic complementation restores appressorium formation and polyurethane degradation in the Δ*pmk1* and Δ*mps1* mutants. (**a**) Molecular confirmation of the complemented strains. "WT" represents the wild-type *A. alternata* FB1. (**b**) Colony morphology of the wild-type, knockout mutant, and complemented strains. (**c**) Restoration of appressorium formation and polyurethane degradation on PU films. "Mycelia" depicts the hyphal morphology of different FB1 complemented strains after 24 h of contact with PU films, with yellow arrows indicating the formation of appressoria structures. "PAUR" and "PEUR" show the macroscopic changes in PAUR or PEUR films, respectively, after three days of co-incubation with each strain. "PEUR-SEM" presents the microstructural morphology of PEUR films observed via SEM after three days of co-incubation with each strain.

In addition, we have expanded the Discussion to consider alternative explanations for the mutant phenotypes. Specifically, we now discuss that the reduced polyurethane degradation observed in the mutants may reflect not only impaired appressorium-mediated penetration but also broader physiological effects, including altered cell-wall

integrity, stress adaptation, secretion, and overall developmental fitness.

The following text has been added to the Discussion section:

"Although the strong correlation between impaired appressorium formation and reduced polyurethane degradation supports a functional role for appressoria in polymer penetration, alternative explanations should also be considered. Pmk1, Mps1, and Msb2 regulate multiple aspects of fungal physiology beyond appressorium development, including cell-wall integrity, stress adaptation, protein secretion, and developmental processes. Therefore, the reduced degradation observed in the corresponding mutants may partly reflect broader physiological defects that indirectly influence polymer colonization and enzyme delivery." (Page 15)

Reviewer #1

Comments to the Authors

This manuscript investigates how the marine fungus Alternaria alternata FB1 degrades polyester and polyether polyurethane, showing that hydrophobic surface sensing via Msb2 activates MAPK/Ca^{2+}-dependent appressorium development and links mechanical penetration with oxidative and hydrolytic depolymerization. The work establishes a mechanistic framework connecting fungal developmental biology with plastic degradation and is conceptually strong and broadly relevant. Several aspects, however, would benefit from clarification and refinement.

Response: Thank you for your comments, we responded to each issue as follows.

Comment 1: Clarify how the PEUR pores observed at 24 h relate to the later degradation outcomes, and explain whether these early micropores act as entry points for enzymatic attack or as sensitizing steps, thereby linking early appressorium activity with subsequent material deterioration.

Response: Thank you for this insightful comment. We agree that the relationship between early pore formation and subsequent polyurethane degradation should be clarified. The manuscript has been revised to explicitly discuss the role of appressorium-induced micropores as an early mechanistic step in the degradation process. We now propose that these pores increase polymer accessibility by exposing internal polymer chains and creating localized entry sites for extracellular oxidases and hydrolases. This initial mechanical disruption likely sensitizes the polymer matrix to subsequent oxidative and hydrolytic depolymerization, ultimately leading to the pronounced changes in molecular weight, thermal properties, and surface morphology observed after prolonged incubation. Corresponding explanations have been added to both the Results and Discussion sections:

"These observations suggest that appressoria-like structures contribute to pore formation through a combination of mechanical penetration and localized enzymatic activity. The resulting micropores may serve as localized entry points for extracellular

enzymes and oxidative reactions, increasing access to internal polymer chains and thereby facilitating the progressive degradation during prolonged incubation." (Page 8)

"The rapid formation of these micropores likely represents a critical initiating step, reflecting appressorium-mediated penetration while simultaneously increasing the accessibility of the polymer matrix to extracellular degradative enzymes. By disrupting surface integrity and exposing internal polymer chains, such localized perforation may facilitate subsequent oxidative and hydrolytic reactions, thereby linking early appressorium activity to the extensive physicochemical changes observed at later stages of degradation." (Page 14)

Comment 2: A clearer distinction between surface sensing and general stress responses would be helpful, particularly by indicating which changes likely reflect early PU recognition and which represent downstream stress adaptation or metabolic reprogramming.

Response: Thanks for your valuable suggestion. We have revised the Results section accordingly. In the revised manuscript, the 6 h PEUR-contact transcriptome is interpreted as an early response associated with hydrophobic surface perception, MAPK and Ca^{2+} signaling activation, and appressorium development, whereas the transcriptional changes observed at 24 h, including alterations in cell-cycle- and DNA replication-related genes, are discussed in the context of subsequent cellular adaptation to prolonged polymer contact. Corresponding revisions have also been made in the Discussion to better distinguish the initial recognition and differentiation processes from later adaptive responses. (Page 8-10, Page 12-13, Page 14-15)

Comment 3: The genetic evidence would be stronger if the lack of complementation were addressed. Because key conclusions rely on the phenotypes of Δmsb2, Δpmk1, and Δ*mps1*, the absence of rescue strains limits causal interpretation. If complementation data are unavailable, this limitation should be acknowledged and the strongest claims moderated.

Response: Thanks for this important suggestion. Complementation analysis

substantially strengthens the causal interpretation of mutant phenotypes. To address this concern, we generated complementation strains for Δ*pmk1* and Δ*mps1* by reintroducing the corresponding wild-type genes. The complemented strains restored appressorium formation upon polyurethane contact and recovered polyurethane degradation activity to levels comparable to those of the wild type, confirming that the observed phenotypes resulted from disruption of the target genes rather than secondary mutations. These new data have been added to the Results section (Page 10).

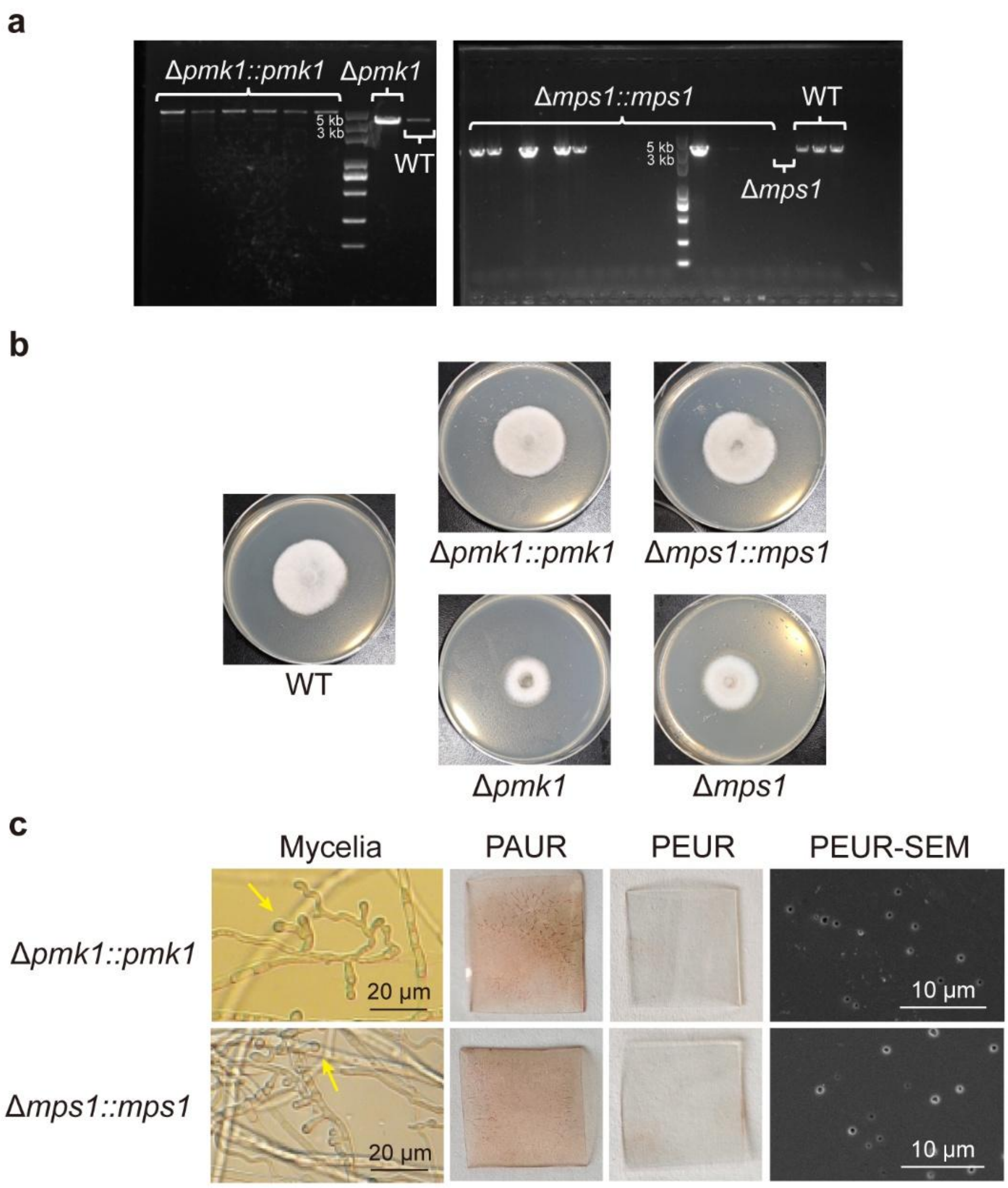

Fig. S5. Genetic complementation restores appressorium formation and polyurethane degradation in the Δ*pmk1* and Δ*mps1* mutants. (**a**) Molecular confirmation of the complemented strains. "WT" represents the wild-type *A. alternata* FB1. (**b**) Colony morphology of the wild-type, knockout mutant, and complemented strains. (**c**) Restoration of appressorium formation and polyurethane degradation on PU films. "Mycelia" depicts the

hyphal morphology of different FB1 complemented strains after 24 h of contact with PU films, with yellow arrows indicating the formation of appressoria structures. “PAUR” and “PEUR” show the macroscopic changes in PAUR or PEUR films, respectively, after three days of co-incubation with each strain. “PEUR-SEM” presents the microstructural morphology of PEUR films observed via SEM after three days of co-incubation with each strain.

We also attempted to generate a complementation strain for Δ*msb2*. However, because of technical difficulties associated with the large size of the Msb2 coding sequence, a stable complemented strain has not yet been obtained. We have therefore moderated the wording of conclusions regarding Msb2 accordingly. Rather than presenting Msb2 as directly mediating polyurethane sensing, the revised manuscript describes it as playing a central role supported by convergent phenotypic, microscopic, calcium-signaling, and transcriptomic evidence.

Comment 4: Please consider discussing alternative explanations for the mutant phenotypes. Reduced degradation in Δ*pmk1*, Δ*mps1*, or Δ*msb2* may reflect not only impaired appressorium-mediated penetration, but also broader defects in secretion, cell-wall integrity, stress tolerance, or overall developmental fitness.

Response: Thank you for your suggestion. We agree that the phenotypes of Δ*pmk1*, Δ*mps1*, and Δ*msb2* may not be solely attributable to defects in appressorium-mediated penetration. Because these regulators are known to control multiple cellular processes, including cell-wall integrity, stress responses, secretion, and fungal development, pleiotropic effects may also contribute to the reduced polyurethane degradation observed in the mutants. To acknowledge this possibility, we have added a discussion of alternative interpretations and study limitations in the Discussion section. While our data support an important role for appressoria in polyurethane degradation, we now explicitly note that additional physiological defects may also influence degradation efficiency.

“Although the strong correlation between impaired appressorium formation and reduced polyurethane degradation supports a functional role for appressoria in polymer penetration, alternative explanations should also be considered. Pmk1, Mps1, and Msb2 regulate multiple aspects of fungal physiology beyond appressorium development,

including cell-wall integrity, stress adaptation, protein secretion, and developmental processes. Therefore, the reduced degradation observed in the corresponding mutants may partly reflect broader physiological defects that indirectly influence polymer colonization and enzyme delivery." (Page 15)

Comment 5: Because the study integrates early transcriptomic and microscopic observations with longer-term degradation assays, providing an overview of the experimental sequence would help readers connect early signaling and morphological events with later material changes.

Response: Thank you for your suggestion. To help readers connect these events, we have added a schematic timeline summarizing the experimental sequence and the corresponding biological processes examined at each stage. The figure highlights the progression from early PU surface sensing and transcriptional responses (6 h), to appressorium formation and pore generation (24 h), mutant phenotyping and initial surface erosion, and finally the physicochemical deterioration of polyurethane during prolonged incubation (7~28 days). This overview provides a temporal framework linking the early cellular responses to the later degradation outcomes.

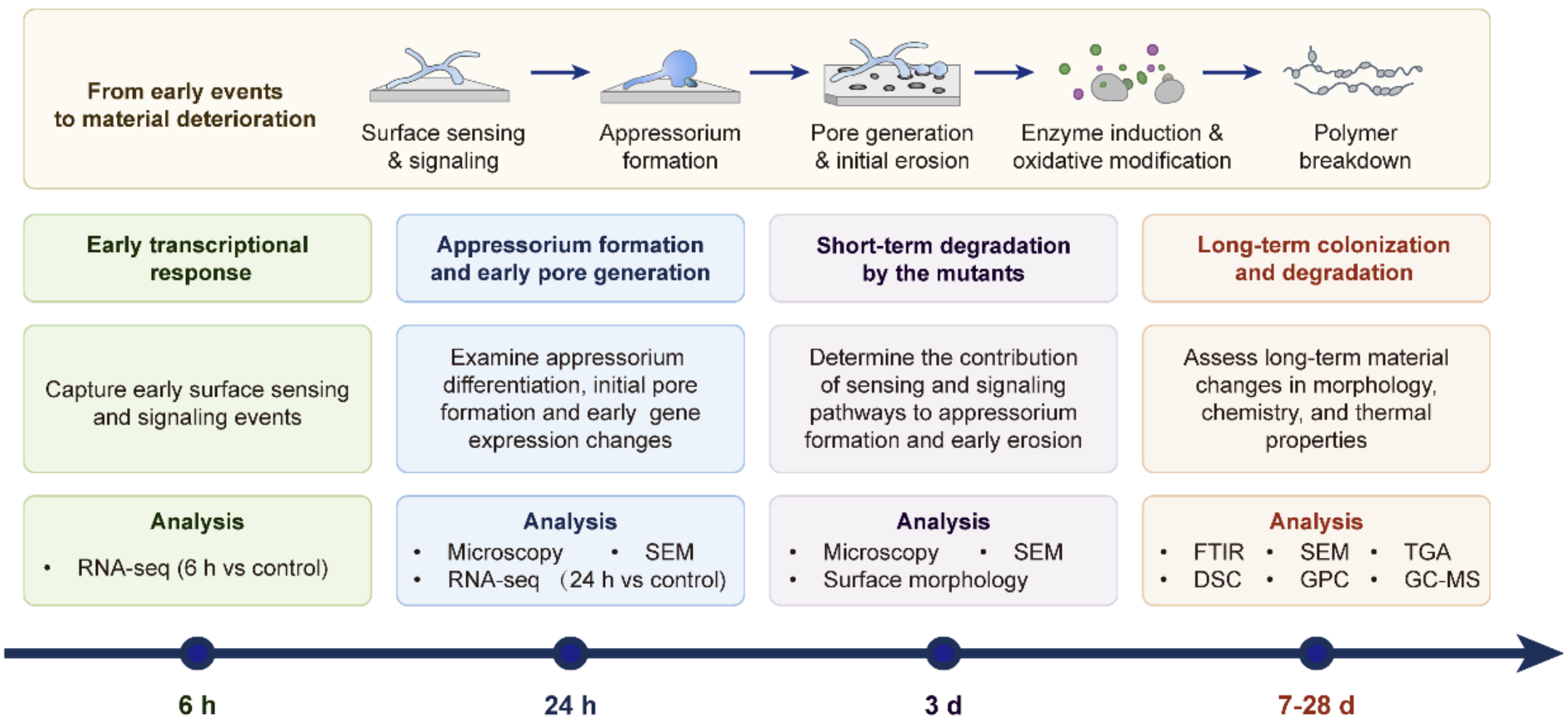

Fig. S10. Schematic timeline of the experimental sequence and the corresponding biological processes.

In addition, we have added the following statement in the Discussion: "Together

with the early transcriptomic and microscopic observations, these findings support a temporal model in which hydrophobic surface sensing initiates appressorium differentiation, pore formation, and activation of degradative pathways, ultimately driving polyurethane deterioration during prolonged incubation.” (Page 14)

Comment 6: Some statements could be slightly softened to avoid overinterpretation. Terms such as “directly mediates,” “principal catalysts,” or “essential” may be stronger than the current evidence supports, particularly where conclusions rely mainly on phenotypic or transcriptomic data.

Response: Thank you for your suggestion. Throughout the revised manuscript, we have carefully moderated the language where conclusions are based primarily on phenotypic observations, transcriptomic analyses, or genetic perturbation experiments. Specifically, terms such as “directly mediates”, “principal catalysts”, “key role”, and “relies on” have been replaced with more cautious expressions. These revisions better reflect the strength of the evidence while preserving the main conclusions of the study.

Comment 7: The manuscript would benefit from light language editing and several overly long sentences could be revised to improve readability and overall flow.

Response: We sincerely appreciate your meticulous and thorough review. We have carefully revised the manuscript to improve readability, sentence structure, and overall flow. Several overly long sentences were shortened or reorganized to enhance clarity, while maintaining the original scientific meaning. We hope this revision meets with your approval.
