# Supplementary material for: Fungal appressoria as mechanochemical organelles for polyurethane degradation
Source: PLoS Genet. 2026 Aug 31;22(8):e1012292. doi: 10.1371/journal.pgen.1012292 (PMC13552942; doi:10.1371/journal.pgen.1012292)
Supplement: Supplementary file 3 [file pgen.1012292.s001.docx]

**S1 Appendix**

**Fungal appressoria as mechanochemical organelles for polyurethane degradation**

Fan Fei^1,2,3,4,5^, Zhenjie Su^1,3,4^, Rui Liu^1,3^, Rongrong Gao^6^, Chaomin Sun^1,3,4*^

^1^Laboratory of Experimental Marine Biology & Center of Deep Sea Research, Shandong Province Key Laboratory of Marine Biodiversity and Bio-resource Sustainable Utilization, Institute of Oceanology, Chinese Academy of Sciences, Qingdao, China

^2^Marine Academy of Zhejiang Province, Hangzhou, China

^3^Laboratory for Marine Biology and Biotechnology, Qingdao Marine Science and Technology Center, Qingdao, China

^4^College of Earth Science, University of Chinese Academy of Sciences, Beijing, China

^5^Key Laboratory of Ocean Space Resource Management Technology, Ministry of Natural Resources, Hangzhou, China

^6^Yantai Vocational College, Yantai, China

^*^ sunchaomin2020@126.com

**Supplementary Methods**

**Fourier-transform infrared spectroscopy (FTIR)**

FTIR spectra were acquired on a Thermo Nicolet iS10 spectrometer (Thermo Scientific, USA) in attenuated total reflectance (ATR) mode. After subtraction of the ambient air background, spectra were recorded over the range 450~4000 cm^-1^ at a resolution of 0.5 cm^-1^, with each sample scanned 32 times to ensure high signal-to-noise ratio.

**Gel permeation chromatography (GPC)**

Molecular‐weight distributions of the films were measured by GPC using an Agilent 1260 Infinity II system (Agilent, USA) equipped with a PL PolarGel-M column (8 µm, 7.5 × 300 mm, Agilent, USA) and a refractive-index detector (RID). Injection volumes were 20 μL, and the flow rate was held at 0.7 mL min^-1^. PEUR samples were eluted with N,N-dimethyl­formamide (DMF) containing 0.1% LiBr at 55 °C, whereas PAUR samples were eluted with tetrahydrofuran (THF) at 35 °C. All samples were dissolved in the corresponding mobile phase overnight, then filtered through a 0.22 µm hydrophobic membrane immediately before injection. Narrow-distribution polystyrene standards (TOSOH, Japan) were used for calibration.

**Thermal stability analyses**

Thermogravimetric analysis (TGA) was performed on a TGA2 apparatus (Mettler Toledo, Switzerland). Approximately 2.5 mg of each film was placed in an alumina crucible and heated under nitrogen at 20 °C min^-1^ from 40 °C to 800 °C, with continuous recording of mass loss to assess thermal decomposition behavior.

Differential scanning calorimetry (DSC) measurements were carried out on a Discovery DSC25 (TA Instruments, USA). Samples of 5~10 mg were loaded into alumina crucibles, covered with lids, and lightly pressed in a DSC pellet press. Under a nitrogen purge, they were heated from 25 °C to 280 °C at 10 °C min^-1^ to determine glass-transition and melting transitions, as well as any crystallization events.

**Gas chromatography-mass spectrometry (GC-MS)**

To identify low-molecular‐weight degradation products generated by strain FB1, PAUR, and PEUR films were incubated in direct contact with fungal mycelia grown on rice solid medium at 28 °C for 28 days. After incubation, mycelia adhering to the film surface and the underlying agar were carefully scraped off and extracted overnight with dichloromethane. The resulting extracts were filtered through a 0.22 µm organic membrane and subjected directly to GC-MS analysis. An Agilent 7890B-5977B GC-MS system fitted with an Agilent J&W DB-5ms column (30 m × 0.25 mm × 0.25 µm) was used, with 1 µL injections in split mode (30:1) at an injector temperature of 280 °C and a carrier-gas flow of 1 mL min^-1^. The GC oven was programmed from 40 °C (2 min hold) to 300 °C at 10 °C min^-1^, followed by a 2 min hold. Mass spectra were recorded over 20~500 amu, with the ion source at 250 °C and the quadrupole at 150 °C.

**Nuclear magnetic resonance (NMR) analysis**

Polyester-based polyurethane samples (5 mg) were dissolved in deuterated dimethyl sulfoxide (DMSO-d_6_) and subjected to proton nuclear magnetic resonance (^1^H NMR) spectroscopy using an Avance spectrometer (Bruker, Germany).

**Pyrolysis-gas chromatography-mass spectrometry (Py-GC/MS) analysis**

Thermal degradation analysis was conducted using a GCMS-QP2010 Ultra gas chromatography-mass spectrometry system (Shimadzu, Japan) equipped with a TG-5SilMS capillary column (30 m × 0.25 mm × 0.25 µm, Thermo Scientific, USA). Approximately 10 mg of each sample was placed into a pyrolysis crucible and directly introduced into the system. The GC oven temperature program was as follows: initial temperature of 60 °C (held for 2 min), ramped at 20 °C/min to 320 °C, and maintained at 320 °C for 13 min. The injection port temperature was set at 320 °C with a split ratio of 100:1. The mass spectrometer operated over an ion scan range of 29~600 amu, with the ion source temperature maintained at 230 °C. The pyrolyzer (PY-3030D, Frontier Lab, Japan) was configured with an interface temperature of 300 °C and a furnace temperature of 550 °C.

**Intracellular calcium quantification**

Hyphae that had not been exposed to the polyurethane film and hyphae that had been in contact with the film for 6 h were collected and washed in PBS. Samples were then incubated with 0.5 µM Fluo-4 AM and Hoechst 33258 staining solution at 28 °C for 15 min. To remove residual AM ester, the loaded aliquots were washed once with PBS containing 2 mM EDTA and then twice with PBS. Fluorescence intensities were measured using a Tecan M200PRO plate reader (Tecan, Switzerland). Fluo-4 was excited at 488 nm with emission recorded at 516 nm, and Hoechst 33258 was excited at 352 nm with emission recorded at 461 nm.

**Supplementary Tables**

**Table A.** **Primers used in this study.**

| **Primers** | **Sequence (5’-3’)** | **Description** |
| --- | --- | --- |
| Upmk1 F | GAGATCGCATCTGGGACTGACTT | To delete gene *pmk1* |
| Upmk1 R | ATGCTCCTTCAATATCAGTTAACGTCGACGTGAAGGTGGGATCTAAGAGCGT | To delete gene *pmk1* |
| Dpmk1 F | CTCGTCCGAGGGCAAAGGAATAGAGATATGGGGAGATGCGCTTCAA | To delete gene *pmk1* |
| Dpmk1 R | TGCATACGATAAGCGATGGTGGA | To delete gene *pmk1* |
| Cd_pmk1 F | TGTCGGCGGTTAGCTCAAAGTA | To verify the deletion of gene *pmk1* |
| Cd_pmk1 R | TGTGGTGAGGAGATTGGCAGAG | To verify the deletion of gene *pmk1* |
| Uhog1 F | AGTAAAAGCGCAGGAAATGGCTG | To delete gene *hog1* |
| Uhog1 R | ATGCTCCTTCAATATCAGTTAACGTCGACGAGTAGGACGTCGTGGGGTATTC | To delete gene *hog1* |
| Dhog1 F | CTCGTCCGAGGGCAAAGGAATAGCTCGAGCTAGAACCATTTGCAGT | To delete gene *hog1* |
| Dhog1 R | GATCTCCACCCGTTCCCAAGTTA | To delete gene *hog1* |
| Cd_hog1 F | GAGACGGGCCCAACAGATAAGA | To verify the deletion of gene *hog1* |
| Cd_hog1 R | GCCCCTGCATATGGTTGTTAGC | To verify the deletion of gene *hog1* |
| Umps1 F | GACGTTACGACTGGCGTTTACTT | To delete gene *mps1* |
| Umps1 R | ATGCTCCTTCAATATCAGTTAACGTCGACAATGGTGGGGTTGGTATCCAGTC | To delete gene *mps1* |
| Dmps1 F | CTCGTCCGAGGGCAAAGGAATAGCAAACCCGGTAACCTACTCGTCA | To delete gene *mps1* |
| Dmps1 R | GCTTCTTCGACTGAGATACGGCT | To delete gene *mps1* |
| Cd_mps1 F | CGTCAACTGTCGGAGAAGGAGT | To verify the deletion of gene *mps1* |
| Cd_mps1 R | GCGAAGACACTCTGGGGTACAT | To verify the deletion of gene *mps1* |
| UpksA F | ACCTCATCCGCTCCGCTCTTACT | To delete gene *pksA* |
| UpksA R | ATGCTCCTTCAATATCAGTTAACGTCGACATCCAGACATCTCCAAGGCTTCGTAC | To delete gene *pksA* |
| DpksA F | CTCGTCCGAGGGCAAAGGAATAGGCGAAGACTGCTTGGCTTGAGGTT | To delete gene *pksA* |
| DpksA R | AGAGGAGTGCCGTTGACCTGGT | To delete gene *pksA* |
| Cd_pksA F | ACAAGGACGCCATCAGGCACCT | To verify the deletion of gene *pksA* |
| Cd_pksA R | CAGGACGTAGCTGCTGGTAGAGGTAG | To verify the deletion of gene *pksA* |
| Ubrm1 F | GCTCATCGTGTGTTCGCAGAGAGA | To delete gene *brm1* |
| Ubrm1 R | ATGCTCCTTCAATATCAGTTAACGTCGACGATCGACGAAGCTGCTGCCAAG | To delete gene *brm1* |

**Table A.** **Primers used in this study (continued).**

| **Primers** | **Sequence (5’-3’)** | **Description** |
| --- | --- | --- |
| Dbrm1 F | CTCGTCCGAGGGCAAAGGAATAGCGGTCGTGATATGTCTGTGTCAAGC | To delete gene *brm1* |
| Dbrm1 R | CCGCTAGGATTTACTTCGCTTGGATT | To delete gene *brm1* |
| Cd_brm1 F | GGAGTTAGGTGCATCAACGATCAAGG | To verify the deletion of gene *brm1* |
| Cd_brm1 R | AGTGCTCGGTTGTGTGGGAGGA | To verify the deletion of gene *brm1* |
| Ubrm2 F | CTCACGTTGTCATACCTGGTGCTTC | To delete gene *brm2* |
| Ubrm2 R | ATGCTCCTTCAATATCAGTTAACGTCGACGGTGGAGGATCGAAGTGTAGAGGT | To delete gene *brm2* |
| Dbrm2 F | CTCGTCCGAGGGCAAAGGAATAGACCGCCGCCTTCATGTCCTT | To delete gene *brm2* |
| Dbrm2 R | CGCCACGCTATGGTACTTCCTTC | To delete gene *brm2* |
| Cd_brm2 F | GACCATGTCGGATCGTCAGGAAC | To verify the deletion of gene *brm2* |
| Cd_brm2 R | ACGAACGGGTCTTGTGTCATAGTGG | To verify the deletion of gene *brm2* |
| Usho1 F | ATCCCTTCAAACGACGTATGTGGCTA | To delete gene *sho1* |
| Usho1 R | ATGCTCCTTCAATATCAGTTAACGTCGACCCAGTGACCAGCAGTATTGAGTAGGC | To delete gene *sho1* |
| Dsho1 F | CTCGTCCGAGGGCAAAGGAATAGGAGAGGGAGGGAAAGAGAACAGCAA | To delete gene *sho1* |
| Dsho1 R | AGTGAATGGAGACGCAGAAGGAGAAA | To delete gene *sho1* |
| Cd_sho1 F | CGCAGTAAGGGAGTGAACGAGGT | To verify the deletion of gene *sho1* |
| Cd_sho1 R | AGAGAGACACACAGAGAGAAGAGTGG | To verify the deletion of gene *sho1* |
| Umsb2 F | GCAGCCTACTTCCACAGCACAGA | To delete gene *msb2* |
| Umsb2 R | ATGCTCCTTCAATATCAGTTAACGTCGACTCCGCCAGTAGAGCCGATGATAGG | To delete gene *msb2* |
| Dmsb2 F | CTCGTCCGAGGGCAAAGGAATAGCAGCACAAGCCGCATGACTTACG | To delete gene *msb2* |
| Dmsb2 R | TGTCCGCAAGCATAGACTGGTATCTT | To delete gene *msb2* |
| Cd_msb2 F | TCCATCCTTGTGCCAACTTCTGA | To verify the deletion of gene *msb2* |
| Cd_msb2 R | GCAGAGGAATCACAAGGTCAACG | To verify the deletion of gene *msb2* |

**Table B.** **BioSample accession numbers and corresponding experimental groups used for RNA-seq analysis.**

| **Experimental group** | **Replicate** | **BioSample ID** |
| --- | --- | --- |
| Control (6 h) | R1 | SAMN49496933 |
| Control (6 h) | R2 | SAMN49496934 |
| Control (6 h) | R3 | SAMN49496935 |
| PEUR-treated (6 h) | R1 | SAMN49496936 |
| PEUR-treated (6 h) | R2 | SAMN49496937 |
| PEUR-treated (6 h) | R3 | SAMN49496938 |
| Control (24 h) | R1 | SAMN49496939 |
| Control (24 h) | R2 | SAMN49496940 |
| Control (24 h) | R3 | SAMN49496941 |
| PEUR-treated (24 h) | R1 | SAMN49496942 |
| PEUR-treated (24 h) | R2 | SAMN49496943 |
| PEUR-treated (24 h) | R3 | SAMN49496944 |
| Δ*pmk1* + PEUR (6 h) | R1 | SAMN49496945 |
| Δ*pmk1* + PEUR (6 h) | R2 | SAMN49496946 |
| Δ*pmk1* + PEUR (6 h) | R3 | SAMN49496947 |
| Δ*osm1* + PEUR (6 h) | R1 | SAMN49496948 |
| Δ*osm1* + PEUR (6 h) | R2 | SAMN49496949 |
| Δ*osm1* + PEUR (6 h) | R3 | SAMN49496950 |
| Δ*mps1* + PEUR (6 h) | R1 | SAMN49496951 |
| Δ*mps1* + PEUR (6 h) | R2 | SAMN49496952 |
| Δ*mps1* + PEUR (6 h) | R3 | SAMN49496953 |
| Δ*msb2* + PEUR (6 h) | R1 | SAMN49496954 |
| Δ*msb2* + PEUR (6 h) | R2 | SAMN49496955 |
| Δ*msb2* + PEUR (6 h) | R3 | SAMN49496956 |
